# Mechanosensitive channel of large conductance enhances the mechanical stretching-induced upregulation of glycolysis and oxidative metabolism in Schwann cells

**DOI:** 10.1186/s12964-024-01497-x

**Published:** 2024-02-01

**Authors:** Fangzhen Shan, Nannan Zhang, Xiaoying Yao, Yi Li, Zihao Wang, Chuanji Zhang, Yuzhong Wang

**Affiliations:** 1 Medical Research Centre, Affiliated Hospital of Jining Medical University, Jining, Shandong Province, China; 2 Department of Respiratory and Critical Care, Affiliated Hospital of Jining Medical University, Jining, Shandong Province, China; 3 Department of Neurology, Affiliated Hospital of Jining Medical University, 89 Guhuai Road, Jining City, Shandong Province 272029 China; 4 Cheeloo Medical College, Shandong University, Jinan, Shandong Province, China; 5 College of Rehabilitation Medicine, Shandong University of Traditional Chinese Medicine, Jinan, Shandong Province, China

**Keywords:** Schwann cells, Mechanosensitive channel of large conductance, Mechanical stretching, Energy metabolism

## Abstract

**Background:**

Physical exercise directly stretching the peripheral nerve promotes nerve regeneration; however, its action mechanism remains elusive. Our present study aimed to investigate the effects of mechanosensitive channel of large conductance (MscL) activated by mechanical stretching on the cultured Schwann cells (SCs) and explore the possible mechanism.

**Methods:**

Primary SCs from neonatal mice at 3–5 days of age were derived and transfected with the lentivirus vector expressing a mutant version of MscL, MscL-G22S. We first detected the cell viability and calcium ion (Ca^2+^) influx in the MscL-G22S-expressing SCs with low-intensity mechanical stretching and the controls. Proteomic and energy metabolomics analyses were performed to investigate the comprehensive effects of MscL-G22S activation on SCs. Measurement of glycolysis- and oxidative phosphorylation-related molecules and ATP production were respectively performed to further validate the effects of MscL-G22S activation on SCs. Finally, the roles of phosphatidylinositol-3-kinase (PI3K)/AKT/mammalian target of rapamycin (mTOR) signaling pathway in the mechanism of energy metabolism modulation of SCs by MscL-G22S activation was investigated.

**Results:**

Mechanical stretching-induced MscL-G22S activation significantly increased the cell viability and Ca^2+^ influx into the SCs. Both the proteomic and targeted energy metabolomics analysis indicated the upregulation of energy metabolism as the main action mechanism of MscL-G22S-activation on SCs. MscL-G22S-activated SCs showed significant upregulation of glycolysis and oxidative phosphorylation when SCs with stretching alone had only mild upregulation of energy metabolism than those without stimuli. MscL-G22S activation caused significant phosphorylation of the PI3K/AKT/mTOR signaling pathway and upregulation of HIF-1α/c-Myc. Inhibition of PI3K abolished the MscL-G22S activation-induced upregulation of HIF-1α/c-Myc signaling in SCs and reduced the levels of glycolysis- and oxidative phosphorylation-related substrates and mitochondrial activity.

**Conclusion:**

Mechanical stretching activates MscL-G22S to significantly promote the energy metabolism of SCs and the production of energic substrates, which may be applied to enhance nerve regeneration via the glia-axonal metabolic coupling.

**Supplementary Information:**

The online version contains supplementary material available at 10.1186/s12964-024-01497-x.

## Background

Schwann cells (SCs), the primary glia in the peripheral nerve, crucially contribute to the functional and structural integrity of the peripheral nervous system (PNS) [1–3]. In addition to forming the myelin, SCs regulate a variety of axonal functions including structural and functional maintenance as well as regeneration after injury via supporting the nutrients and energic substrates [4, 5]. In the case of peripheral nerve injury, SCs dedifferentiate, migrate to the injured site, clear the nerve debris, and then redifferentiate for further remyelination [6, 7]. Recent progress has revealed the transfer of metabolic substrates from SCs to axons, namely glial-axonal metabolic coupling, as the main energy source of axons in the PNS [8, 9]. Under the stimulus, especially after the nerve injury, SCs will take the glycolytic shift to adapt to the high energy demand, which is mainly modulated by the metabolic signaling pathway, including phosphatidylinositol-3-kinase (PI3K), AKT, and mammalian target of rapamycin (mTOR) [8–10]. The glycolysis deficiency and mitochondrial dysfunction of SCs ablate axonal survival and will cause the decline of peripheral nerve function [11, 12]. Metabolic interaction between SCs and axons has been applied in understanding the physiological and pathological mechanism of peripheral neuropathy and regulation of glial-axonal metabolic coupling has been emerging as a new strategy for these diseases.

Physical exercise directly stretching the peripheral nerve mechanically serves as one of the most helpful treatments for recovery of peripheral neuropathy. Particularly, although wildly applied, the therapeutic efficiency of physical exercise is usually slow clinically and the action mechanism of mechanical stretching on peripheral nerve regeneration remains elusive [13, 14]. Recently, one possible mechanism of mechanical stretching on peripheral nerve regeneration via stimulating the mechanoresponsive components of the cellular membrane, which sense physical stimulation and initiate intracellular signaling pathways, has been brought up [15, 16]. A variety of mechanically sensitive proteins have been identified to display significant potential for modulating neural activity and growth under mechanical stimulus [17–19]. Several mechanosensitive channels endogenously expressed on SCs, including transient receptor potential channels and Piezo channels, have been identified, which can be activated by mechanical stimulation to modulate the differentiation and myelination of SCs [20–22]. Mechanosensitive channel of large conductance (MscL), the first exclusively identified mechanosensitive ion channel from *Escherichia coli*, acts as a release valve of cytoplasmic permeation when membrane tension rises by various mechanical stimuli, such as mechanical stretching and ultrasound [23, 24]. In response to the tension conveyed via the lipid bilayer, MscL increases its open probability and functions effectively in any cell membrane, including cultured neurons [14, 17]. Being different from other mechanosensitive proteins, MscL responds to the membrane tension without the requirement of other intracellular proteins or ligands [17, 25, 26]. Soloperto et al. demonstrated that the engineered expression of MscL increases neuronal sensitivity to mechanical stimulation while preserving the physiological development of the neuronal network [25]. Ye et al. reported a mutant version of MscL (I92L) activated by surface acoustic waves facilitates the controlling of rat hippocampal neuron excitation in vitro [17]. Recently, Qiu et al. reported that low-intensity ultrasound activating MscL-G22S, which is ~ 30% easier to open than wild-type MscL, significantly promoted the calcium ion (Ca^2+^) influx and neuron activation in vitro [26]. Given that activation of MscL promotes the membrane permeability to yield the exchange of substances inside and outside the cell, it remains unknown whether MscL activated by mechanical stretching affects neuron excitation via modulation of energy metabolism.

In this study, we demonstrated that MscL-G22S activated by mechanical stretching significantly promoted Ca^2+^ influx and increased energy metabolism in cultured SCs through the PI3K/AKT/mTOR pathway and its downstream hypoxia-inducible factor 1-alpha (HIF-1α)/c-Myc signaling. We also found that MscL-G22S activation robustly elevates glucose uptake, glycolysis, and oxidative phosphorylation as well as the mitochondrial activity in SCs. Our findings provide the basic evidence that engineered expression of MscL in SCs with mechanical stretching may serve as a potential strategy for peripheral nerve regeneration via the upregulation of the energy metabolism of SCs.

## Materials and methods

### Cell culture

Primary SCs were prepared from the sciatic nerves of C57BL/6 mice aged 3–5 days as previously described [27]. Briefly, after removal of the epineurium, sciatic nerves were cut into small slices and then digested at 37 °C in 0.05% collagenase/dispase (Roche, 10,269,638,001, Mannheim, Germany) for 1 h, followed by digestion using 0.25% trypsin (Gibco, 25,200,056, NY, USA) for 15 min. Dissociated cells were resuspended in complete culture medium consisting of DMEM/F12 medium, 10% fetal bovine serum, 10 ng/mL heregulin-β-1 (Peprotech, 100–03, NJ, USA), 2 μM forskolin (Sigma, F6886, MA, USA) and 1% penicillin/streptomycin, and then planted in cell plates pre-coated with poly L-lysine and laminin. After 48 h of culture, half of the culture medium was replaced with fresh culture medium containing 10 μM Ara-C (MCE, HY-13605, NJ, USA) for further culture of 2 days. The media was then switched to a complete culture medium for 2 days for further cell growth. The cell passage was digested with 0.125% trypsin to remove fibroblasts. A purity of SCs greater than 95% identified by immunostaining of S100β and p75 neurotrophin receptor permits the subsequent experiments. Our study was approved by the Ethics Committee of Medical Science Research of the Affiliated Hospital of Jining Medical University (Reference number, 2022B005).

### Plasmid construction and transfection

The mutant *E. coli* MscL-G22S cDNA was synthesized by Sangon Biotech Co., Ltd. (Shanghai, China) and subsequently ligated into a lentiviral vector of pLentai-hEF1a-PuroR-mCherry with a CMV promoter. The plasmid map is shown in Fig. S1. The lentiviral plasmids were co-transfected with helper plasmids into HEK293T cells to package the lentivirus (Taitool Bioscience Co., Ltd., Shanghai, China). For the MscL-G22S expression, SCs were transfected with the lentivirus according to the manufacturer’s instructions. pLentai-hEF1a-PuroR-MCV-mCherry was used as the negative control for the MscL-G22S expression. After transfection, puromycin (2 μg/mL) was added into the culture medium from the second passage to screen the resistant cells. For mitochondrial membrane potential (MMP) measurement, MscL-G22S was amplified using primers containing NheI and EcoRI cut sites and subcloned into a modified pcDNA3.1 vector (Invitrogen, CA, USA) and the MscL-G22S-pcDNA3.1 plasmid were transfected into SCs using EntransterTM-H4000 (Engreen, 4000–5, Beijing, China) according to the manufacturer’s instructions. Expression of MscL-G22S was detected using quantitative reverse transcription polymerase chain reaction (qRT-PCR), western blot, and immunostaining.

### Mechanical stretching

To investigate the effects of mechanical stretching-induced MscL-G22S, four groups of SCs with different treatments were set as follows, MscL-G22S-expressing SCs with mechanical stretching (MscL-G22S activation), MscL-G22S-expressing SCs without stretching, SCs with mechanical stretching, and SCs without treatment (neither stretching nor MscL expression). The SCs were seeded in a BioFlex® six-well plate (Flexcell International Corporation, NC, USA) with laminin-precoated elastic membranes for the stimulus of mechanical stretching at 0 ~ 6% strain and 0.2 Hz for 24 h using the Flexcell® FX 6000™ Tension System (Flexcell International Corporation, NC, USA) inside the regular incubator (37 °C, 5% CO_2_). For the un-stretched controls, SCs seeded in the same BioFlex® plates were maintained in an identical manner but in the absence of any membrane tension. After a culture of 24 h, the cell viability was measured in a microplate reader (BioTek Cytation Hybrid, Agilent, CA, USA) using cell counting kit-8 (CCK-8, Dojindo, CK04, MD, USA) according to the manufacturer’s instructions. To investigate the role of PI3K in the action mechanism of MscL-G22S activation on SCs, a potent inhibitor of PI3K, LY294002 (MEC, HY-10108, NJ, USA) was added into the culture of MscL-G22S-expressing SCs (10 mM) before mechanical stretching in some experiments.

### Proteome analysis

To investigate the effects of MscL-G22S activation on cultured SCs, Tandem Mass Tags proteome quantitative analysis was performed by PTM BioLab (Hangzhou, China) using liquid chromatography-mass spectrometry. In brief, after 24 h of mechanical stretching, 5 × 10^6^ SCs were collected and sonicated three times on ice using a high-intensity ultrasonic processor in a lysis buffer. After centrifugation at 12,000 g for 20 min at 4 °C, the protein concentration of the supernatants was determined by bicinchoninic acid assay. The protein solution was then digested into peptides using trypsin, labeled with Tandem Mass Tags reagent (Thermo Fisher Scientific, Rockford, IL, USA), and fractionated into fractions by high pH reverse-phase HPLC using Agilent 300 Extend C18 column according to manufacturer’s protocol. Subsequently, the peptides were separated using EASY-nLC 1200 UPLC (Thermo Fisher Scientific) and analyzed in Q ExactiveTM HF-X (Thermo Fisher Scientific) with a nano-electrospray ion source. Data was collected using a data-dependent acquisition procedure and processed using the MaxQuant search engine (v.1.6.15.0). False positive rate was adjusted to < 1%.

Differentially expressed proteins (DEPs) were identified based on fold change > 1.2 and Student’s t-test with *p* value < 0.05. Cluster membership was visualized by a heat map using the “heatmap.2” function from the “gplots” R-package. UniProt-GOA was used to generate Gene Ontology (GO) annotation. Proteins were classified by GO annotation into three categories: biological process, cellular compartment, and molecular function. Kyoto Encyclopedia of Genes and Genomes (KEGG) database was used to analyze enriched pathways. Wolfpsort subcellular localization prediction soft was used to predict subcellular localization. Only significant GO and KEGG terms with *p* value < 0.05 were considered.

### Quantification of cellular metabolites by mass spectrometry

To investigate the effects of MscL-G22S activation on energy metabolism of cultured SCs, targeted energy metabolomics was performed by Metware Biotechnology (Wuhan, China) using liquid chromatography-mass spectrometry. In brief, after being stretched for 24 h, 2 × 10^6^ SCs were collected and mixed with 500 μL of 80% methanol/water (precooled at − 20 °C) and vortexed at 2500 rpm/min for 2 min. The samples were frozen in liquid nitrogen for 5 min and then vortexed using a homogenizer. After an incubation at − 20 °C for 30 min, the samples were centrifuged at 12,000 g for 10 min at 4 °C. The supernatant was collected and subjected to an AB-SCIEX QTRAP 6500 mass spectrometer. The peak area and retention time were extracted using multiquanta software and normalized to standard energy metabolizers for metabolite identification. Significantly regulated metabolites between groups were determined by fold change (≥ 2 or ≤ 0.5) and variable importance in projection (≥ 1).

### Measurement of intracellular Ca^2+^

SCs were loaded with the fluorescent calcium indicator Fluo-4 AM (Beyotime, S1061, Beijing, China) before mechanical stretching according to the manufacturer’s instructions. Briefly, cells were washed three times with PBS and incubated with 2 μM Fluo-4 AM in the dark for 30 min at 37 °C. Then, cells were washed three times with PBS and cultured in a buffer solution with 130 mM NaCl, 2 mM MgCl_2_, 4.5 mM KCl, 10 mM Glucose, 20 mM HEPES, and 2 mM CaCl_2_, pH 7.4. An Axio observer 7 microscope (Zeiss, Jena, Germany) was used to capture the fluorescence images with emission of 520 nm at different time points before and after mechanical stretching as follows, 0 min, 5 min, 10 min, 30 min, 1 h, 6 h, 12 h, and 24 h. At least 10 visual fields per group cells were captured. The fluorescence intensity was quantitatively analyzed by ImageJ software (NIH, MD, USA).

### qRT-PCR

Total RNA was extracted and purified from cultured cells using TRIzol reagent (Invitrogen, 15,596,026, CA, USA). Then reverse transcription of RNA to cDNA using PrimeScript™ II 1st Strand cDNA Synthesis Kit (Takara, 6210A, Beijing, China). RT-qPCR was performed on a CFX96 Touch™ system (Bio-Rad, CA, USA) using UltraSYBR Mixture (Cwbio, CW0957, Taizhou, China). According to the Ct value of the target gene, the relative expression of the target gene was calculated with a comparative quantitative method for 2^−ΔΔCt^ and normalized to the housekeeping gene, glyceraldehyde-3-phosphate dehydrogenase (GAPDH). For the mitochondrial DNA (mtDNA copy number) assay, total DNA was extracted from SCs using a Genomic DNA Kit (Tiangen, DP304, Beijing, China) as the template for qRT-PCR. The mtDNA copy number was determined by using mitochondrially encoded subunit 2 of cytochrome c oxidase and further normalized to the nuclear ribosomal protein s18 as previously described [28]. The primers used for PCR are listed in Supplemental Table 1.

### Western blot

Total proteins were extracted from SCs using RIPA buffer supplemented with 1 mM PMSF (Solarbio, P0100, Beijing, China) and protease inhibitor cocktail (MCE, Y-K0012, NJ, USA). Protein samples were loaded on SDS-PAGE gels for electrophoresis and then transferred to PVDF membranes. After blocking using 5% bovine serum albumin in tris-buffered saline with 0.1% Tween® 20 detergent, the membrane was incubated with the diluted primary and secondary antibodies, respectively. The reaction was visualized with chemiluminescence. Details of primary and secondary antibodies used for western blot were listed in Supplemental Table 2.

### Immunofluorescence

After culture, SCs were fixed in 4% paraformaldehyde (Solarbio, P1110, Beijing, China) in phosphate-buffered saline (PBS) for 20 min and then permeabilized using 0.3% of TritonX-100 in PBS containing 10% normal goat serum for 30 min at room temperature. Following three times with PBS, SCs were incubated with primary antibodies diluted in PBS containing 5% normal goat serum at 4 °C overnight. After washing, SCs were further incubated with fluorescent-labeled secondary antibodies and iFluor™ 647-phalloidin (Yeasen Biotechnology, 40762ES75, Shanghai, China) for 2 h at room temperature. Following three washing, cells were mounted with anti-fadent mountant solutions containing 4′, 6-diamidino-2-phenylindole (Solarbio, Beijing, China) and finally imaged using Axio Imager Z2 microscope (Zeiss, Jena, Germany). At least 10 visual fields per group cells were captured. Details of primary and secondary antibodies are listed in Supplemental Table 2.

### Transmission electron microscopy

The cells for electron microscopy investigation were performed by a modified procedure as described previously [29]. Briefly, SCs were fixed with 2.5% glutaraldehyde and 4% paraformaldehyde in PBS for 6 h. After a post-fixation in aqueous 1% osmium tetroxide for 1 h, the cell samples were dehydrated in a gradient concentration of alcohol and embedded in epoxy resin. 70 nm thickness sections were prepared using an ultramicrotome (RMC-PT-PC, Tucson, AZ, USA) and stained with 2% uranyl acetate and 1% lead citrate. The ultrastructure observation was performed using a Tolas L 120C electron microscopy (Thermo Fisher Scientific, Brno, Czech). The length and average area of mitochondria were determined using ImageJ software. At least 10 visual fields per group cells were captured for the statistical analysis.

### MMP measurement

The MMP measurement of SCs was performed using a JC-1 Kit (Abcam, ab113850, MA, USA). Briefly, after mechanical stretching, the cells were washed with PBS and then incubated with the JC-1 working solution in the dark for 10 minutes at 37 °C. After two washing using PBS, the cells were added with a culture medium and placed in a Cell Discoverer 7 (Zeiss, Jena, Germany). The emission wavelengths of 590 nm and 529 nm were used to monitor the aggregate and monomeric JC-1, respectively. The fluorescence intensity of at least 10 visual fields per group cells was quantified using the ImageJ software. The ratio of aggregated/monomeric JC-1 was used to quantify changes in the MMP of SCs with different treatments.

### Measurement of energy metabolism

Oxygen consumption rate (OCR) and extracellular acidification rates (ECAR) were measured using a Seahorse XFe24 analyzer (Agilent Technologies, CA, USA). After 24 h of mechanical stretching, SCs were subjected to the poly-L-lysine-coated Seahorse XFe24 plates (80,000 cells per well) for a culture of 6 h for the adherent. Then, the SCs were added with Seahorse XF DMEM (Agilent Technologies, 103,575–100, CA, USA) without fetal bovine serum for further incubation without CO_2_ at 37 °C for 1 h. Three baseline measurements were recorded before applying different compounds. For OCR, oligomycin (1.5 mM), carbonyl cyanide-4 (trifluoromethoxy) phenylhydrazone (FCCP, 2 μM), and rotenone/antimycin A (0.5 μM each) were sequentially added to measure the basal respiration, coupling efficiency, and spare respiratory capacity. For ECAR, glucose (10 mM), oligomycin 1.5 μM), and 2-deoxy-glucose (2-DG, 50 mM) were sequentially added to measure the glycolysis rate, glycolytic capacity, and glycolytic reserve. Real-time ATP production rate from glycolysis and mitochondria respiration was measured using a Seahorse XF real-time ATP rate assay kit (Agilent Technologies, 103,592–10). ATP measurements were recorded followed by the injection of oligomycin (1.5 mM) and rotenone/antimycin A (0.5 μM each), respectively. The data was analyzed using Wave Desktop 2.6 software (Agilent Technologies).

### Nicotinamide adenine dinucleotide (NAD^+^ and NADH) measurement

The levels of NAD^+^ and NADH were determined using a WST-8-based NAD^+^/NADH assay kit (Beyotime, S0175, Beijing, China). In brief, after mechanical stretching, 1 × 10^6^ SCs were washed twice with cold PBS and further lysed using NADH/NAD extraction buffer. The samples were centrifuged at 12,000 g for 10 min to collect the supernatant. For the measurement of total NAD^+^/NADH, 20 μL of cell lysis supernatant was added with 90 μL of working solution for incubation in the dark at 37 °C for 10 min. Then, 10 μL of color-developing agent was added to the plate for further incubation at 37 °C for 30 min. The absorbance was measured at 450 nm using a microplate reader (BioTek Cytation Hybrid, Agilent, CA, USA). For the measurement of NADH, the supernatant was incubated at 60 °C for 30 min first and then processed into the procedure as described above. The levels of NAD^+^ were derived by subtracting NADH from total NAD^+^/NADH.

### Measurement of glucose, glucose-6-phosphate, pyruvate and lactate

Glucose uptake was analyzed using the fluorescent glucose analog, 2-[N-(7-nitrobenz-2-oxa-1,3- diazol-4-yl)amino]-2-deoxy-D-glucose (2-NBDG) (Abcam, ab204702, MA, USA). Cells in BioFlex® six-well plates were washed with PBS and incubated in glucose-free culture medium for 1 h at 37 °C. Subsequently, 2-NBDG was added to reach a final concentration of 100 μM, and the cells were further incubated for 1 h under mechanical stretching. After the treatment, cells were collected and suspended in a cell-based assay buffer. The levels of 2-NBDG uptake were determined using flow cytometry (Beckman, DxFLEX, CA, USA) at 488 nm.

The levels of glucose-6-phosphate, lactate, and pyruvate were measured using high-sensitivity assay kits (for glucose-6-phosphate, Sigma, MAK021, Taufkirchen, Germany; for pyruvate, Abcam, ab65342, MA, USA; for lactate, Abcam, ab65330, MA, USA) according to the manufacturer’s instructions, respectively. The absorbance was recorded using a microplate reader at 450 nm for glucose-6-phosphate and at 570 nm for lactate and pyruvate.

## Statistical analysis

Statistical comparison between two or more groups of data was respectively performed by two-tailed Student’s *t*-test or one-way analysis of variance followed by Bonferroni post-test using GraphPad Prism 9 (GraphPad Software, CA, USA). For all bar graphs, representative data from at least three independent experiments were shown as mean ± standard deviation. A two-sided *p* value of < 0.05 was considered significant.

## Results

### MscL-G22S activation increases the cell viability of SCs

**Fig. 1:**
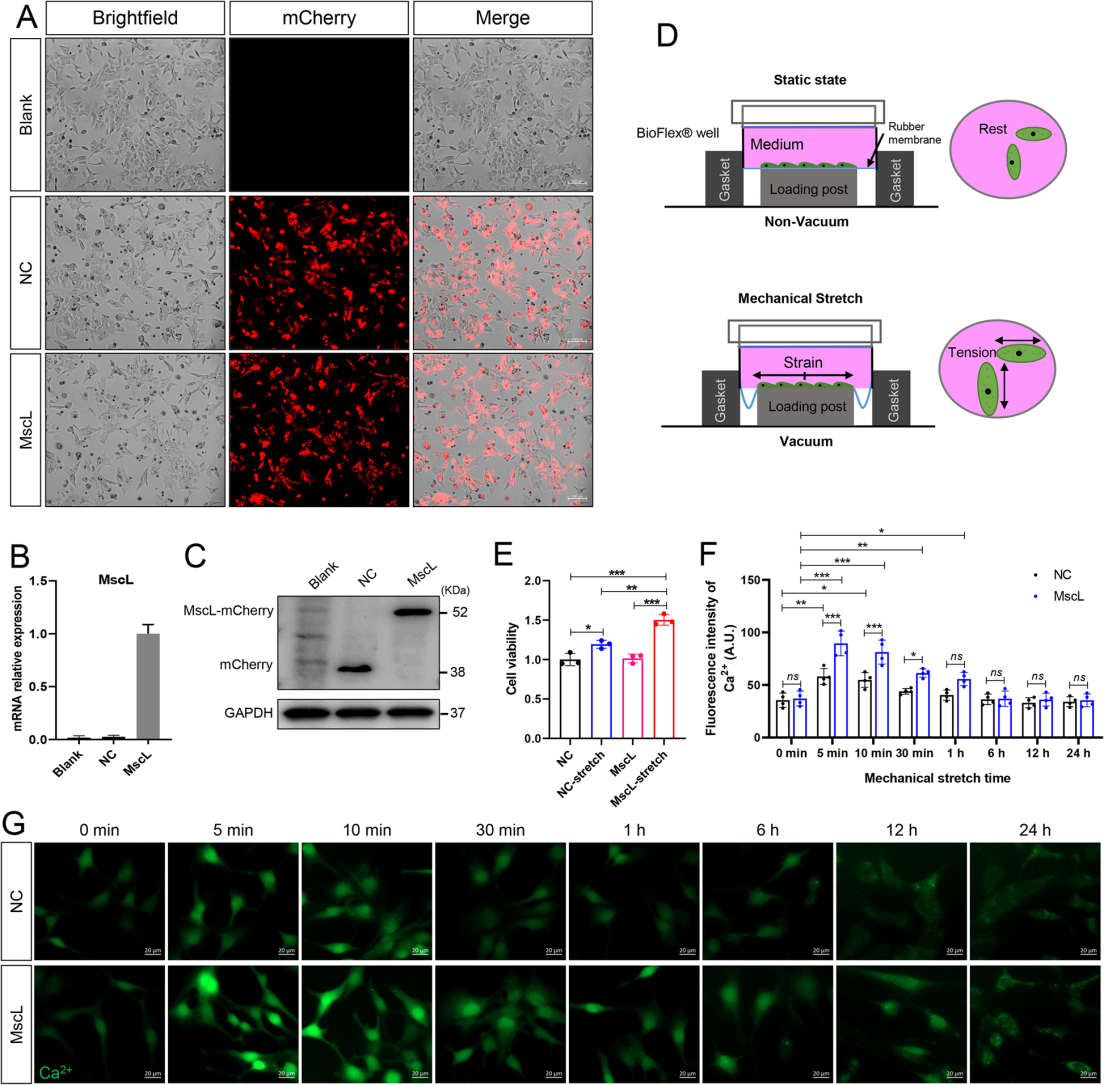
Mechanical stretching increases the cell viability and Ca^2+^ influx in MscL-G22S-expressing SCs. **A**-**C**. Expression of MscL-G22S in cultured SCs (“MscL”) transfected with a lentiviral vector encoding the mCherry and MscL-G22S was detected by immunofluorescence, qRT-PCR and western blot. SCs transfected with negative-vector control (NC) and those without transfection (Blank) were used as the controls. For the western blot, detection of MscL-G22S is visualized by IgG antibodies against the mCherry. **D**. The SCs with or without MscL-G22S expression were mechanically stretched using a Flexcell Tension system. **E**. CCK-8 assay showed that MscL-G22S activated by mechanical stretching significantly increased the viability of SCs. **F**-**G**. Quantitative analysis and representative fluorescence images of intracellular Ca^2+^ stained using Fluo-4 AM at indicated time points before and after mechanical stretching. Scale bars = 20 μm. NC, negative control; MscL, MscL-G22S-expressing SCs; NC-stretch, negative control with mechanical stretching; MscL-stretch, MscL-G22S-expressing SCs with mechanical stretching. *ns*, not significant, *p < 0.05, **p < 0.01, ***p < 0.001

MscL-G22S has a lower activation pressure threshold than the wild-type MscL but does not exhibit spontaneous activity [25, 30]. The expression of MscL-G22S in SCs was verified by qPCR, western blot and immunostaining (Fig. 1A-C). As shown in Fig. 1D, MscL-G22S-expressing SCs or their controls were mechanically stretched using the Flexcell Tension system. CCK-8 assay demonstrated that MscL-G22S-expressing SCs with mechanical stretching had significantly higher viability than those without stretching and SCs without MscL-G22S expression. Furthermore, SCs with mechanical stretching also had higher viability than those without stretching (Fig. 1E).

Ca^2+^ is a universal second messenger that participates in a series of physiological processes of neuron and glial cells including depolarization, synaptic activity, axon growth and regeneration as well as energy metabolism [26, 31–33]. As shown in Fig. 1F and H, MscL-G22S activation triggered a significant persistent Ca^2+^ influx in cultured SCs up to 30 mins after mechanical stretching than those in the controls.

### Proteomic profiling reveals that MscL-G22S activation upregulates lipid synthesis, glycolysis, and OXPHOS in SCs

**Fig. 2:**
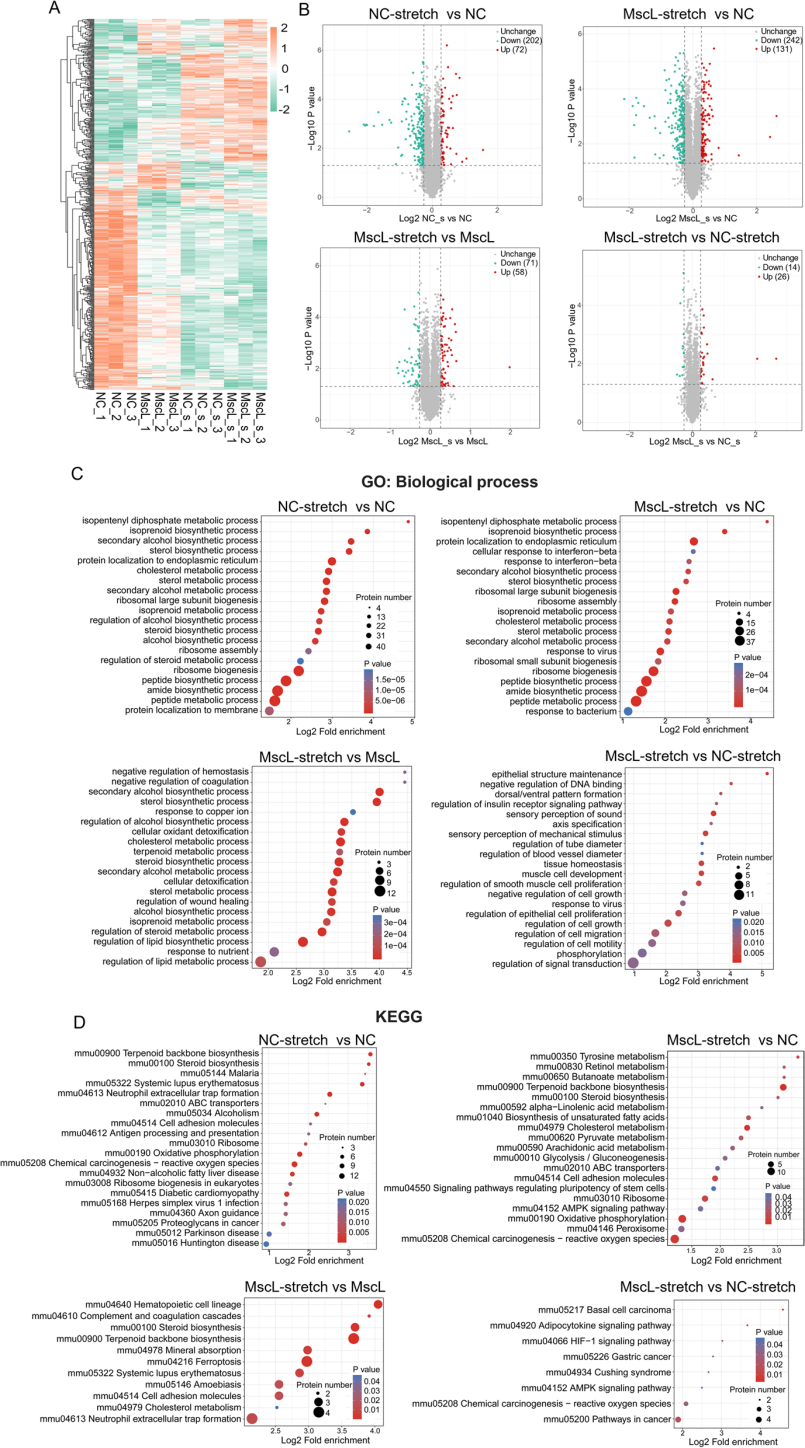
Proteomic analysis of the effects of MscL-G22S activation on energy metabolism in SCs. Data of proteomic profiles analysis of SCs with different treatments were shown as heat maps, volcano plot, Gene ontology (GO) and Kyoto Encyclopedia of Genes and Genomes (KEGG) enrichment analysis. **A** The scaled expression value (row Z score) is shown as an orange-green color scheme where orange suggests for higher expression and green suggests for lower expression. **B** A series of differentially expressed proteins (DEPs) were identified between SCs with different treatment (fold change ≥1.2 or ≤ − 1.2; *p* < 0.05). In the plot, “down” means down-regulated DEPs, “up” means up-regulated DEPs and “unchange” means the difference was not statistically significant. The GO (**C**) and KEGG enrichment analysis (**D**) showed that the DEPs among SCs with different treatments were associated a variety of biological process, especially the biosynthesis and metabolism. NC, negative control; MscL, MscL-G22S-expressing SCs; NC-stretch, negative control with mechanical stretching; MscL-stretch, MscL-G22S-expressing SCs with mechanical stretching

The proteomic analysis revealed a total of 7334 DEPs in SCs with different treatments. Hierarchical clustering and Principal-component analysis (PCA) of the DEPs almost entirely separated MscL-G22S-activated SCs from those with mechanical stretching alone, with MscL-G22S expression and without treatment (Fig. 2A and Fig.S2A), suggesting a significant association between DEPs and MscL-G22S activation as well as the mechanical stretching. As shown in Fig. 2B and Fig. S2B, 40 DEPs were identified between MscL-G22S-activated SCs and those with mechanical stretching alone, which are mainly located in the plasma membrane, nucleus, cytoplasm, extracellular and mitochondria. 123 DEPs between MscL-G22S-activated SCs and MscL-G22S-expressing controls mainly located in the nucleus, cytoplasm, extracellular, plasma membrane and mitochondria. For the other DEPs between SCs with different treatments, the details are shown in Fig. S2B.

The results of GO and KEGG pathway enrichment analysis showed that the DEPs between MscL-G22S-activated SCs and those with stretching alone were associated with adipocytokine signaling pathway, HIF-1 signaling pathway, AMPK signaling pathway, sensory perception of the mechanical stimulus, transmembrane signaling receptor activity, transcription cofactor binding, and cell motility, migration and growth (Fig. 2C-D and Fig. S2C-D). The DEPs between MscL-G22S-activated SCs and MscL-G22S-expressing controls were related to lipid synthesis including terpenoid, steroid, cholesterol and arachidonic acid biosynthesis, oxidoreductase activity (Fig. 2C-D and Fig. S2C-D). For the SCs with or without mechanical stretching, the DEPs were related to lipid synthesis including terpenoid, steroid and cholesterol biosynthesis, OXPHOS, ribosome assembly and biogenesis, ATP-binding cassette transporter, cell adhesion molecules and axon guidance (Fig. 2C-D and Fig. S2C-D). Overall, in the cultured SCs, MscL-G22S activation caused significant changes in series of protein expression in the nucleus, cytoplasm, membrane, and mitochondria, which upregulates lipid synthesis, glycolysis, OXPHOS, pyruvate metabolism, ribosome biogenesis, HIF-1 signaling pathway and sensory perception of mechanical stimulus. Furthermore, mechanical stretching alone also induces significant changes in nuclear protein expression to regulate the transcription and translation of cytoplasm, membrane, and mitochondria-associated proteins, resulting in an upregulation of lipid synthesis, OXPHOS, and ribosome biogenesis.

### MscL-G22S activation altered energy-related metabolic pathways

**Fig. 3:**
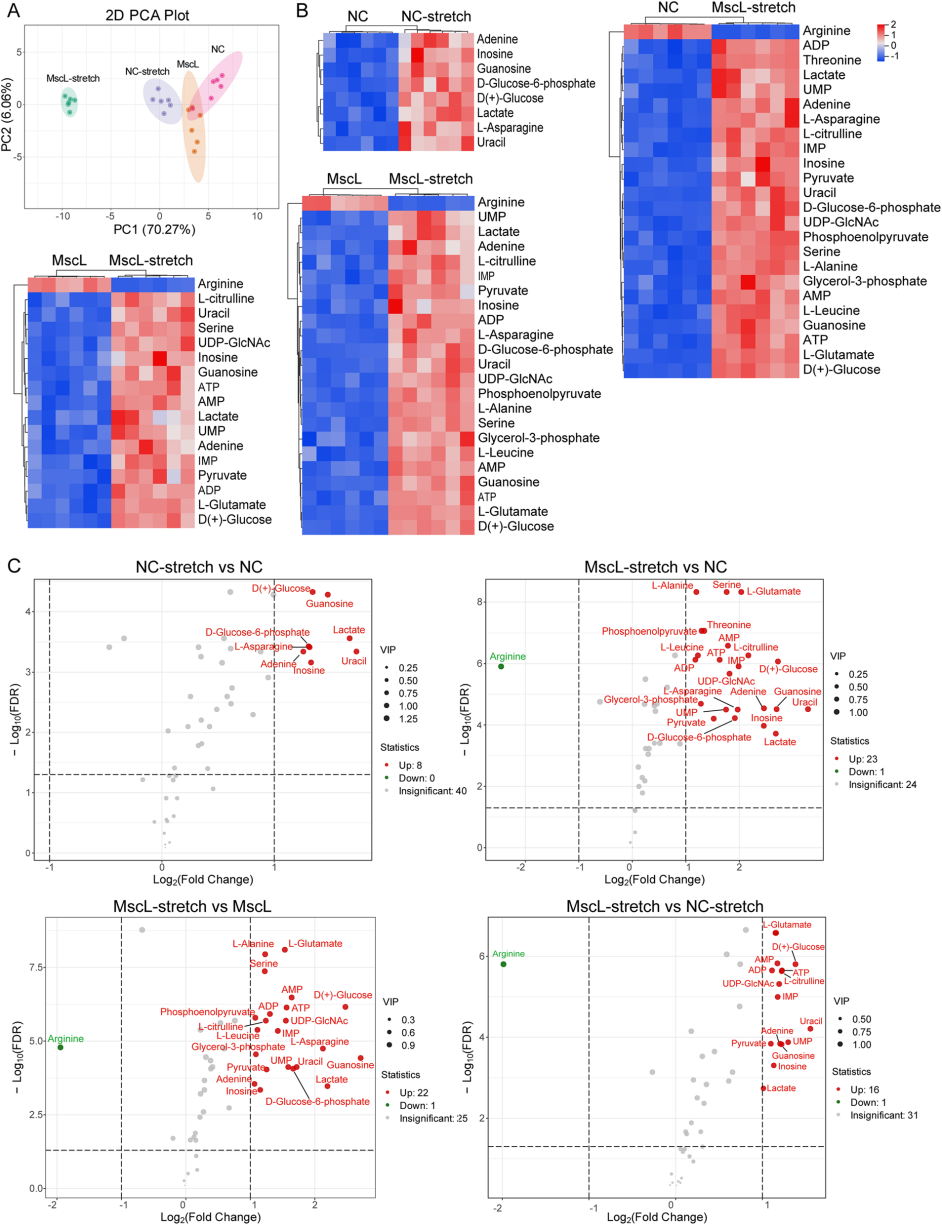
Metabolomic analysis of the effects of MscL-G22S activation on energy metabolism in SCs. **A** Total principal-component analysis (PCA) was conducted on overall samples to preliminarily understand the overall metabolic differences among samples and the variation degree among samples within the group. The PCA results showed the separation trend of metabolome among each group, indicating differentially expressed substrates in metabolome between sample groups. The heatmap (**B**) and volcanic map (**C**) showed significant upregulation of metabolites in SCs with MscL-G22S activation than the controls. NC, negative control; MscL, MscL-G22S-expressing SCs; NC-stretch, negative control with mechanical stretching; MscL-stretch, MscL-G22S-expressing SCs with mechanical stretching. The comparison in energy metabolite analysis between MscL-G22S-activated SCs and other controls were shown in Fig. S4-S7

Because the proteomic analysis has identified a significant association between MscL-G22S activation and energy metabolism-related processes in cultured SCs, we then investigated the effects of MscL-G22S activation on the energy-related metabolic pathways of SCs using targeted energy metabolomics analysis. PCA was conducted on the 48 identified metabolites (Fig. 3A). The scores plot showed a distinct separation of the sample groups, indicating obvious differences in metabolomes in different groups. Metabolites that were significantly up-or down-regulated in different groups (with a fold change cutoff of > 2.0 or < 0.5, *p* < 0.05, VIP > 1) were subject to pathway analysis.

The clustered heatmap and volcano plot of significant difference metabolites in the energy metabolism pathways are shown in Fig. 3B-C. For the SCs without MscL-G22S expression, mechanical stretching significantly increased the levels of D(+)-glucose, D-Glucose 6-phosphate, L-asparagine, guanosine, uracil, inosine, adenine and lactate (Fig. S3A). The pathways of glycolysis, purine and pyrimidine metabolism, pentose phosphate pathway, pantothenate, CoA biosynthesis, and pyruvate metabolism were enriched based on the above six metabolites. Our data demonstrated that mechanical stretching alone could also upregulate glycolysis and purine/pyrimidine metabolism (Fig. S3B).

For MscL-G22S-expressing SCs, mechanical stretching significantly increased the levels of glucose uptake and glycolytic metabolites (glucose 6-phosphate, glycerol 3-phosphate, phosphoenolpyruvate, pyruvate, lactate) when compared with the SCs without MscL-G22S expression, and SCs without mechanical stretching (Fig. S4A and Fig. S5A). Similarly, we observed significantly higher levels of amino acids (L-alanine, serine, L-leucine, L-asparagine, L-citrulline, L-glutamate, and threonine) involved in the TCA cycle in MscL-G22S-activated SCs. In addition, we found a greater abundance of purine and pyrimidine metabolites (Adenine, guanosine, uracil, inosine, IMP, UMP, AMP, ADP, ATP, etc.), which is crucial for nucleotide synthesis and energy supply in MscL-G22S-activated SCs. Pathway enrichment analysis based on differential metabolites identified several major pathways including glycolysis, purine and pyrimidine metabolism, pyruvate metabolism, TCA cycle, amino acids metabolism, and lipid metabolism (Fig. S4B and Fig. S5B). When compared to SCs with mechanical stretching alone, the levels of glucose, glycolytic metabolite (pyruvate, lactate), and amino acids (serine, L-citrulline, L-glutamate) involved in TCA cycle, purine and pyrimidine metabolites (Adenine, guanosine, AMP, ADP, ATP, etc.) were significantly increased in MscL-G22S-activated SCs (Fig. S6A). The pathways of purine and pyrimidine metabolism, glycolysis, pyruvate metabolism, TCA cycle and amino acids metabolism were enriched based on the differential metabolites (Fig. S6B). Besides, the abundance of arginine was significantly decreased in MscL-G22S-activated SCs in comparison to the other controls, which may be caused by the conversion of arginine to citrulline in the urea cycle and further supplying materials for the TCA cycle. The metabolic phenotypes were demonstrated to be consistent with the abundance of proteins related to the pyruvate-lactate axis and OXPHOS. Collectively, these findings demonstrate that mechanical stretching alone could slightly upregulate the levels of glycolysis, whereas MscL-G22S activation by mechanical stretching simultaneously up-regulates the levels of glycolysis, TCA, and OXPHOS in SCs.

### MscL-G22S activation increases the glycolysis and oxidative phosphorylation (OXPHOS) in SCs

**Fig. 4:**
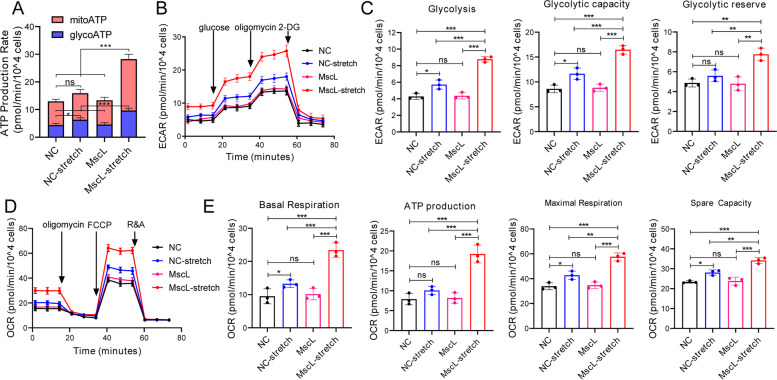
MscL-G22S activation increases the glycolysis and OXPHOS in SCs. **A** Mitochondrial and glycolytic ATP production rates were measured using real-time ATP Rate Assay. **B**-**E** Extracellular and acidification rate (ECAR) and mitochondrial oxygen consumption ratio (OCR) were determined by Seahorse experiments (**B**, **D**). Quantitative analysis of glycolysis rate, glycolytic capacity, and glycolytic reserve in ECAR (**C**) and basal respiration, ATP production, maximal respiration, and spare respiratory capacity in OCR (**E**) demonstrated that the levels of energy metabolism were significantly upregulated in MscL-G22S-expressing SCs after mechanical stretching when compared to the controls. 2-DG, 2-deoxy-glucose; FCCP, carbonyl cyanide 4-(trifluoromethoxy) phenylhydrazone; R&A, rotenone/antimycin A; NC, negative control; MscL, MscL-G22S-expressing SCs; NC-stretch, negative control with mechanical stretching; MscL-stretch, MscL-G22S-expressing SCs with mechanical stretching. *ns*, not significant, *p < 0.05, **p < 0.01, ***p < 0.001

Both the proteomic and targeted energy metabolomics analysis suggested that MscL-G22S activation by mechanical stretching affects the SCs via modulation of energy metabolism. Glycolysis and OXPHOS serve as two of the major energy-producing mechanisms in cells. In this study, we measured the ATP production rate of SCs using Agilent Seahorse XFe24 Analyzers with a real-time ATP rate assay kit. As shown in Fig. 4A, we observed a significantly higher rate of both glycolytic and mitochondrial ATP production in MscL-G22S-activated SCs than in the control SCs. SCs with mechanical stretching only showed a higher rate of glycolytic ATP production but not mitochondrial ATP production than those without stretching. Moreover, extracellular acidification rate (ECAR) and oxygen consumption rate (OCR) were detected to monitor the glycolysis and OXPHOS of SCs, respectively. The results showed that the levels of glycolysis rate, glycolytic capacity, and glycolytic reserve in ECAR of MscL-G22S-activated SCs were significantly higher than those of the controls (Fig. 4B-C), and there were only higher levels of glycolysis rate and glycolytic capacity in SCs with mechanical stretching only than those without stretching (Fig. 4B-C). The basal respiration, as well as ATP production, maximal respiration, and respiratory capacity in OCR were also significantly increased in MscL-G22S-activated SCs than the controls (Fig. 4D-E). SCs with mechanical stretching only showed higher basal respiration, maximal respiration, and respiratory capacity than those without stretching (Fig. 4D-E). These results indicated that mechanical stretching-induced MscL-G22S activation remarkedly increased the glycolysis and OXPHOS in SCs while mechanical stretching alone had mild effects on the energy metabolism of the cultured SCs.

### MscL-G22S activation upregulated glycolysis-related genes in SCs

**Fig. 5:**
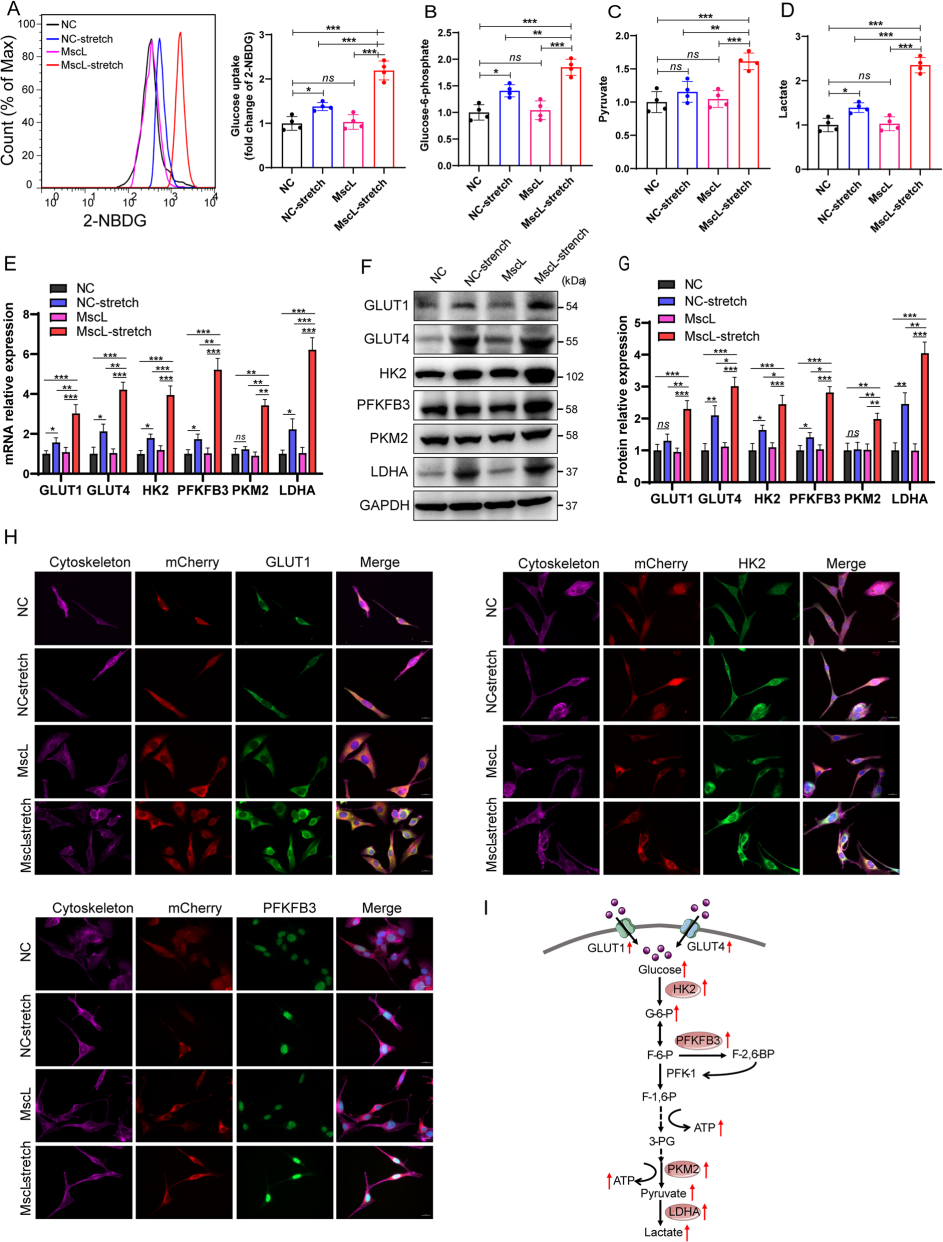
Mechanical stretching upregulates glycolysis in MscL-G22S-expressing SCs. Under the mechanical stretching, the levels of glycolysis in MscL-G22S-expressing SCs were measured by detecting the glucose uptake, glycolytic products, and related enzymes. As shown, there were significantly higher levels of glucose uptake (**A**), glucose-6-phosphate (**B**), pyruvate (**C**), and lactate (**D**) in MscL-G22S-expressing SCs with mechanical stretching than the controls. Both the real-time PCR (**E**) and western blot (**F**-**G**) analysis showed significantly higher levels of GLUT1, GLUT4, HK2, PFKFB3, PKM2, and LDHA in MscL-G22S-activated SCs than the controls. **H** Immunofluorescence staining showed the upregulated glycolysis-related proteins in MscL-G22S-activated SCs. The quantitative data of immunofluorescence is shown in Fig. S8. **I** A schematic diagram highlights the effects of MscL-G22S activation on the up-regulation (red arrows) of key proteins and glycolytic products in SCs. Scale bars: 20 μm. The data shown were representative of at least three independent experiments. NC, negative control; MscL, MscL-G22S-expressing SCs; NC-stretch, negative control with mechanical stretching; MscL-stretch, MscL-G22S-expressing SCs with mechanical stretching. *ns*, not significant, *p < 0.05, **p < 0.01, ***p < 0.001

As the primary step of glucose metabolism to extract energy for the cellular physiological process, glycolysis is regulated by a series of catalytic enzymes and factors. After transport of glucose through the cell membrane, which depends on a family of eight developmental regulated glucose transporters (GLUTs) [34], glucose is phosphorylated by hexokinase (HK) into glucose-6-phosphate, which is further presented into the process regulated by 6-phosphofructo-2-kinase/fructose-2,6-biphosphatase 3 (PFKFB3), a key glycolysis-regulatory enzyme [35]. After several steps of reaction, the generated phosphoenolpyruvate is catalyzed by pyruvate kinase to form pyruvate [36], which can be further processed into the tricarboxylic acid (TCA) cycle or converted into lactate by lactate dehydrogenase A (LDHA) [37]. In our study, MscL-G22S-activated SCs showed significantly higher levels of glucose uptake, intracellular glucose-6-phosphate, pyruvate, and lactate than the other control SCs (Fig. 5A-D). Then, we examined the changes in expression of glycolytic-related genes in SCs with different treatments. MscL-G22S-activation caused significantly higher mRNA levels of HK2, PFKFB3, pyruvate kinase M2 (PKM2), and LDHA in SCs than in the controls (Fig. 5E). Both western blot and immunostaining analysis also demonstrated that the protein expression of HK2, PFKFB3, PKM2, and LDHA was significantly increased in MscL-G22S-activated SCs than the controls (Fig. 5F-H and Fig. S7A-B). Being consistent with the increase of glucose uptake, the mRNA and protein expression levels of GLUT isoforms 1 and 4 (GLUT1/GLUT4) were also significantly higher in MscL-G22S-activated SCs than those in the controls (Fig. 5E-H and Fig. S7A-B). When compared to the control without stimulus, SCs with mechanical stretching also had significantly higher levels of GLUT4, PFKFB3 and LDHA, indicating relatively mild effects of mechanical stretching alone on the glycolysis of cultured SCs. Altogether, these results demonstrated that MscL-G22S activation by mechanical stretching remarkedly increased the levels of glycolysis and upregulated the expression of glycolysis-related genes (Fig. 5I).

### MscL-G22S activation promotes the OXPHOS via upregulation of mitochondrial activity in SCs

**Fig. 6:**
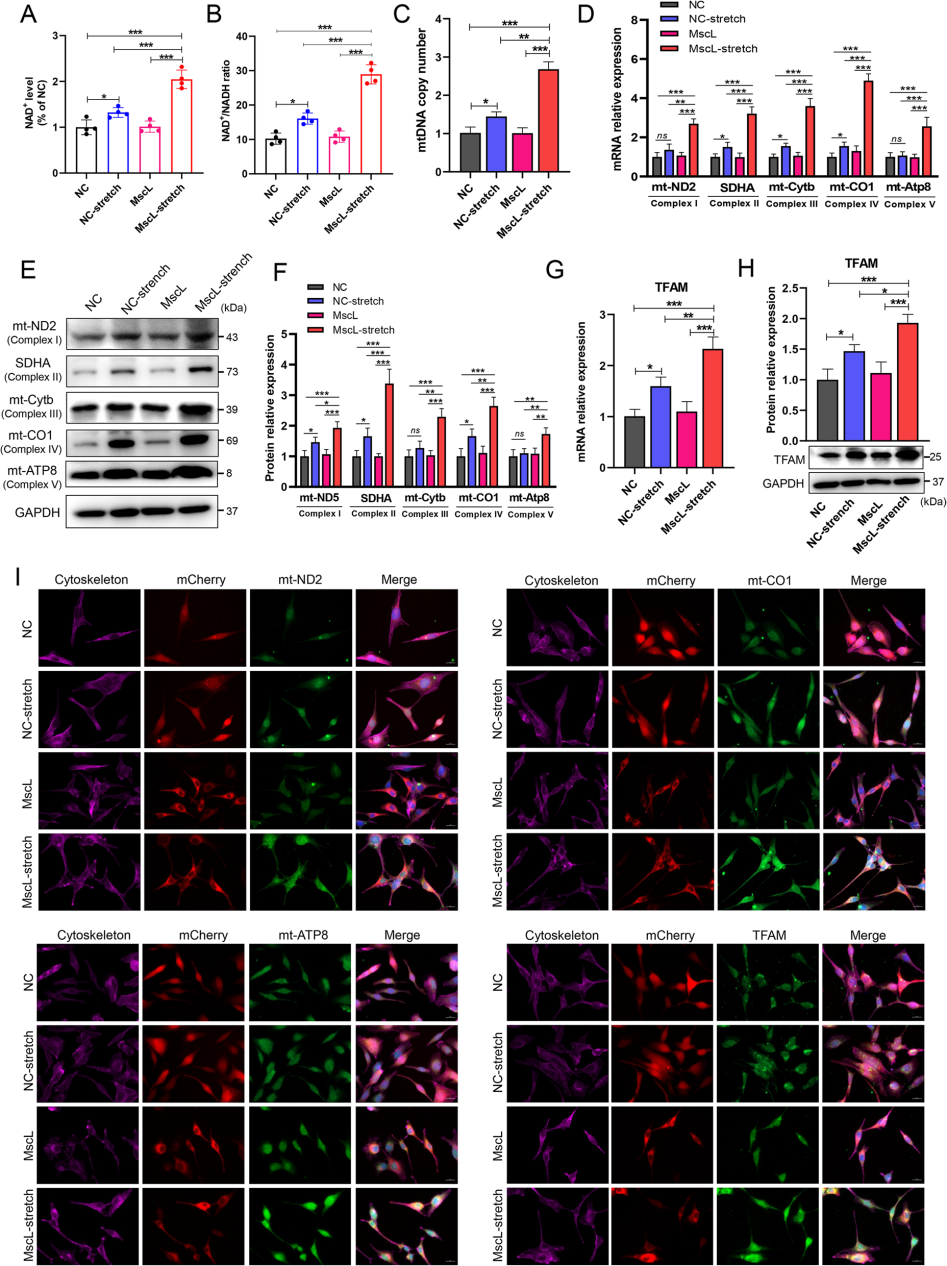
Mechanical stretching upregulates OXPHOS in MscL-G22S-expressing SCs. The effects of MscL-G22S activation by mechanical stretching on OXPHOS of SCs were evaluated by analysis of the levels of NAD^+^ (**A**), NAD^+^ ratio to NADH (**B**), mtDNA copy number (**C**), expression of mitochondrial electron transfer chain components, including mt-ND2, SDHA, mt-Cytb, mt-CO1 and mt-Atp8 (**D** for real-time PCR, **E** and **F** for western blot). When compared to the controlled SCs, there were significantly higher levels of NAD^+^, NAD^+^ ratio to NADH, and upregulated mitochondrial electron transfer chain components in MscL-G22S-activated SCs. **G**-**H** Both the mRNA and protein expression of TFAM were significantly upregulated in MscL-G22S-activated SCs. **I** Immunostaining of mitochondrial electron transfer chain proteins and TFAM in SCs was shown. The quantitative data of immunofluorescence is shown in Fig. S9. Scale bars: 20 μm. The data shown were representative of at least three independent experiments. NC, negative control; MscL, MscL-G22S-expressing SCs; NC-stretch, negative control with mechanical stretching; MscL-stretch, MscL-G22S-expressing SCs with mechanical stretching. *ns*, not significant, *p < 0.05, **p < 0.01, ***p < 0.001

NAD^+^, a key carrier molecule for electrons and hydrogen, becomes NADH when two electrons and hydrogen are added to the molecule, which plays a critical role in the OXPHOS-associated reactions [38]. Under the stimulus of mechanical stretching, MscL-G22S-expressing SCs had significantly higher levels of NAD^+^ and ratio of NAD^+^/NADH than the other controls (Fig. 6A-B). The mitochondrial respiration depends on mtDNA, which encodes proteins for respiratory chain complexes and their nuclear-coding components. We measured the mtDNA copy number and mRNA expression of mitochondrial electron transport chain (ETC) components, including mt-ND2, SDHA, mt-Cytb, mt-Co1, and mt-Atp8 in SCs with different treatments. The PCR analysis demonstrated that there was significantly greater mtDNA copy number and higher mRNA levels of mt-ND2, mt-Cytb, mt-CO1, mt-Atp8, and SDHA in MscL-G22S-activated SCs than the control SCs (Fig. 6C-D). Both western blot and immunostaining analysis also demonstrated that the protein expression levels of mt-ND2, SDHA, mt-Cytb, mt-CO1, and mt-Atp8 were significantly upregulated in MscL-G22S-activated SCs than the controls (Fig. 6E-F, I and Fig. S8A-B). Mitochondrial transcription factor A (TFAM) plays a role in modulating the mtDNA copy number and mitochondrial-encoded components of the respiratory complexes [39]. We also observed significantly higher mRNA and protein expression of TFAM in MscL-G22S-activated SCs than in the controls (Fig. 6G-I and Fig. S8B). When compared to the controls without stimulus, SCs with mechanical stretching also had significantly higher levels of SDHA, mt-Cytb, mt-CO1, mtDNA copy number and TFAM, indicating relatively mild effects of mechanical stretching alone on the OXPHOS of cultured SCs.

**Fig. 7:**
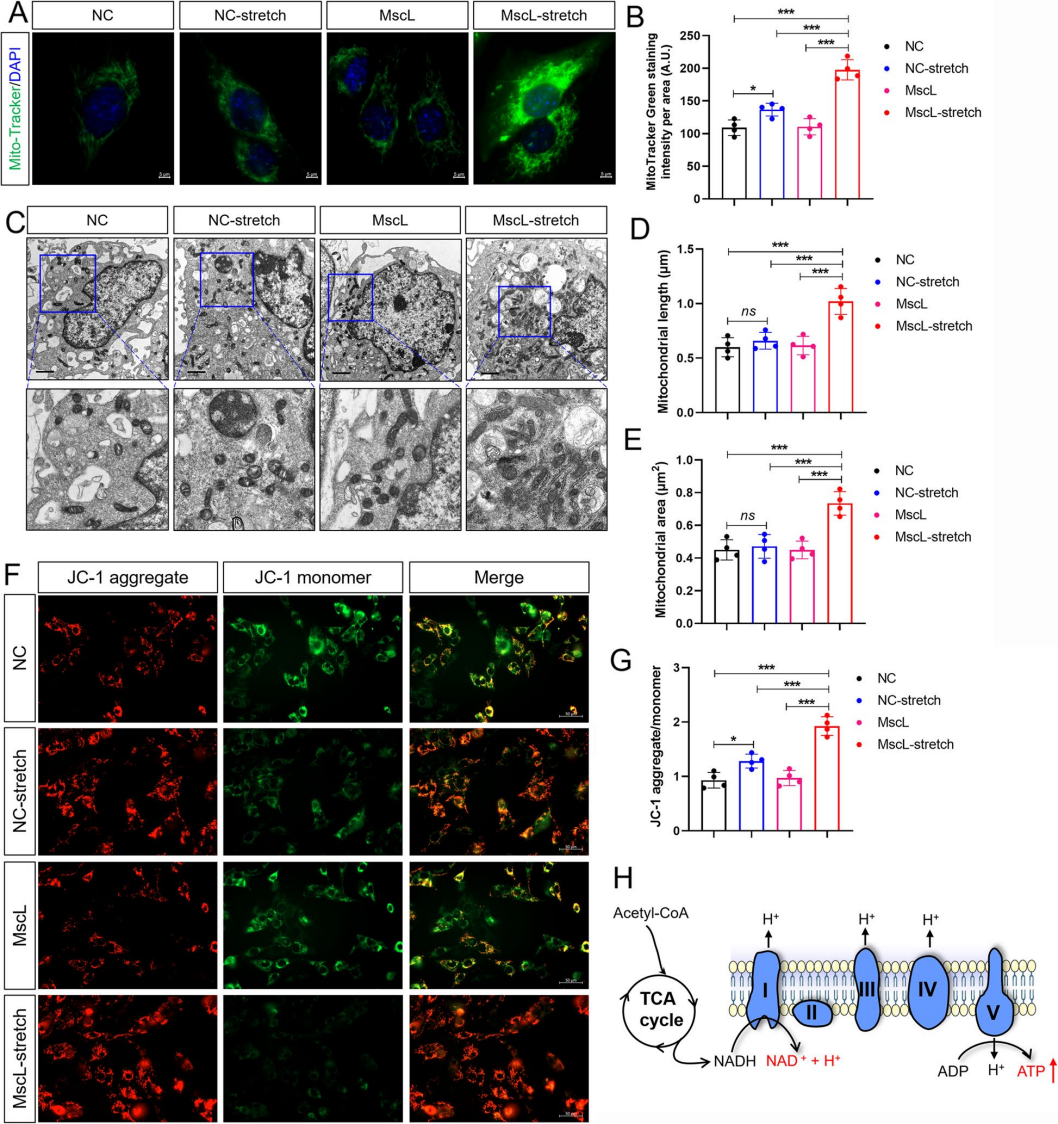
Mechanical stretching increases the number and activity of mitochondria in MscL-G22S-activated SCs. **A**-**B** The mitochondria in SCs were stained with MitoTracker Green (Scale bars = 5 μm). As indicated, there was significantly higher mitochondrial intensity, indicating the greater number of mitochondria, in MscL-G22S-activated SCs. **C**-**E** The data of electron microscopy showed significantly greater mitochondrial length and area in MscL-G22S-activated SCs than the controls (Scale bars = 2 μm). **F**-**G** Mitochondrial membrane potential (MMP) in SCs was measured by JC-1 staining to evaluate the mitochondrial activity (Scale bars = 50 μm). JC-1 aggregates indicate high MMP while the JC-1 monomer indicates low MMP. Quantification analysis of the ratio of JC-1 aggregate/monomer showed significantly increased mitochondrial activity in MscL-G22S-activated SCs than the controls. **H** a schematic diagram annotates the effects of mechanical stimuli on the mitochondrial respiration of MscL-G22S-expressing SCs, including upregulated expression of ETC complexes, greater mitochondrial number, area, and MMP as well as the increased production of ATP. The data shown were representative of four independent experiments. At least 10 visual fields of cultured SCs with different treatments were captured for each biological replicate. NC, negative control; MscL, MscL-G22S-expressing SCs; NC-stretch, negative control with mechanical stretching; MscL-stretch, MscL-G22S-expressing SCs with mechanical stretching. *ns*, not significant, *p < 0.05, **p < 0.01, ***p < 0.001

To further confirm the effects of MscL-G22S activation on mitochondria, the length, area, and density of mitochondria were respectively investigated using electron microscope and immunostaining. Our data showed that MscL-G22S-expressing SCs following mechanical stretching had significantly greater length, area, and number of mitochondria than the control SCs (Fig. 7A-E). MMP generated by proton pumps of the ETC is required for ATP production. Our data of JC-1 fluorescence analysis showed a significant increase of MMP in MscL-G22S-expressing SCs following mechanical stretching than the other controls (Fig. 7F-G). SCs with mechanical stretching only showed a greater number of mitochondria with increased MMP than the controls without stretching while there was no difference in the length and area of mitochondria between SCs with and without stretching (Fig. 7A-G). Overall, these results suggested that MscL-G22S activation by mechanical stretching significantly increased both the number and activity of mitochondria in SCs (Fig. 7H).

### MscL-G22S activation increased the energy metabolism of SCs through the PI3K/AKT/mTOR pathway

**Fig. 8:**
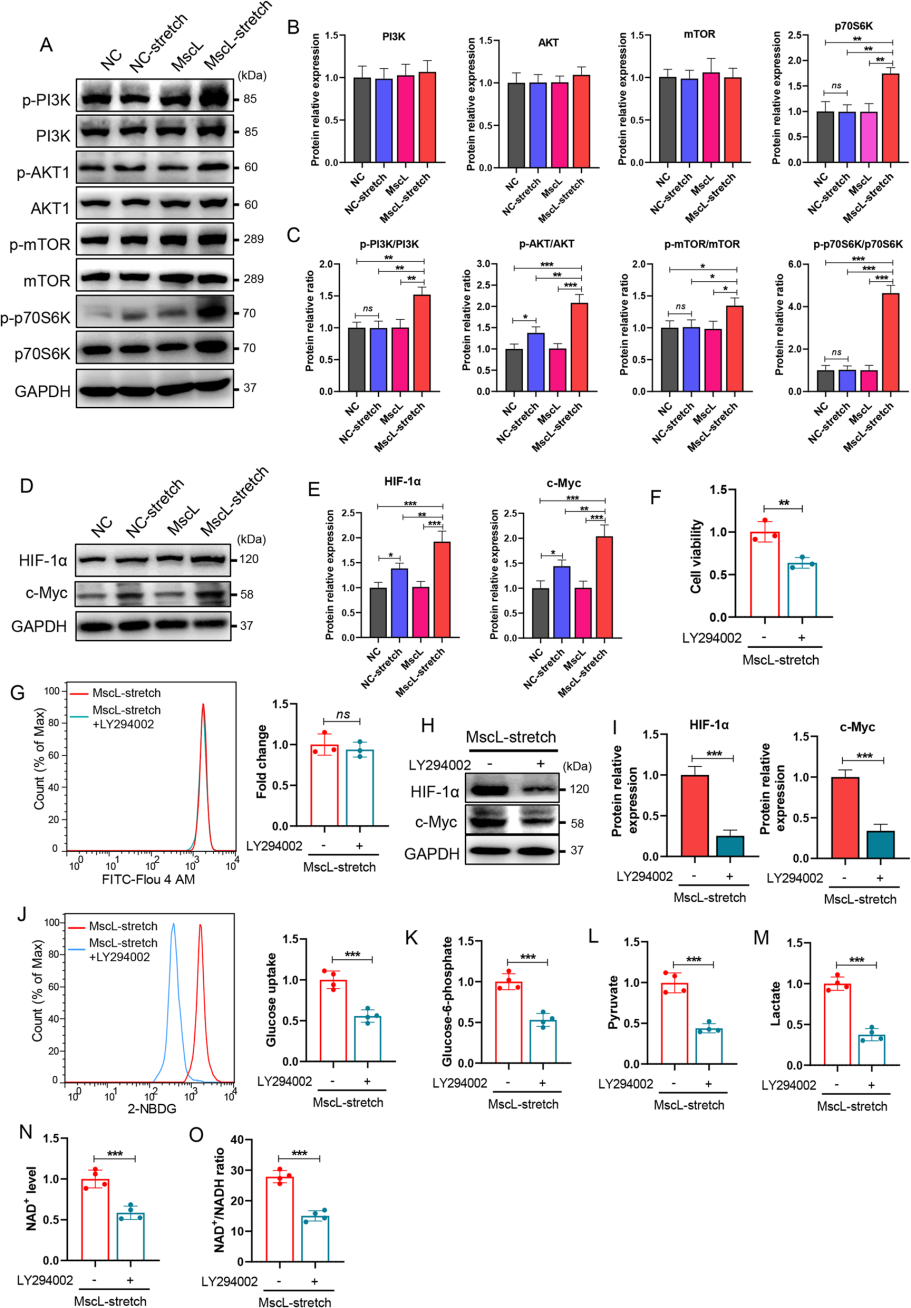
MscL-G22S activation increases the energy metabolism of cultured SCs through the PI3K/AKT/mTOR pathway. **A**-**C** PI3K/AKT/mTOR pathway contributes to the regulation of cellular metabolism. The western blot analysis showed significantly higher phosphorylated levels of the PI3K, AKT, mTOR, and their downstream molecule, p70S6K, in MscL-G22S-activated SCs than the controls. Notably, higher levels of phosphorylated Akt but not the PI3K, mTOR and p70S6K were observed in stretched SCs than those without stimulus, indicating relatively mild effects of mechanical stretching only on cultured SCs. **D**-**E** Mechanical stretching induced significant upregulation of HIF-1α and c-Myc expression in cultured SCs, especially for the SCs with MscL-G22S expression. **F**-**O** LY294002, a potent PI3K inhibitor, was used to further validate the roles of PI3K signaling pathway in MscL-G22S activation-induced upregulation of energy metabolism in cultured SCs. As indicated, inhibition of PI3K significantly reduced the MscL-G22S activation-induced upregulation of cell viability but did not affect the Ca^2+^ influx in the SCs with mechanical stretching for 10 min (**F**-**G**). Similarly, after a culture of 24 h, expression of HIF-1α and c-Myc, the levels of glucose uptake, glucose-6-phosphate, pyruvate and lactate (**H**-**M**), NAD^+^ and NAD^+^/NADH ratio (**N**-**O**) in MscL-G22S-activated SCs were significantly reduced by inhibition of PI3K. The data shown were representative of at least three independent experiments. NC, negative control; MscL, MscL-G22S-expressing SCs; NC-stretch, negative control with mechanical stretching; MscL-stretch, MscL-G22S-expressing SCs with mechanical stretching. *ns*, not significant, *p < 0.05, **p < 0.01, ***p < 0.001

Because Ca^2+^ and its downstream PI3K/AKT/mTOR pathway participate in the cellular energetic processes [8, 40] and we have demonstrated mechanical stretching significantly promoted the Ca^2+^ influx into MscL-G22S-expressing SCs, we further investigated the effects of MscL-G22S activation on the PI3K/AKT/mTOR pathway in SCs. Although there were no differences in protein expression of PI3K/AKT/mTOR pathway in SCs with different treatment, significantly higher phosphorylation levels of PI3K (on Y607), AKT (on S473), and mTOR (on S2448) was observed in MscL-G22S-activated SCs (Fig. 8A-C). Previous studies demonstrated p70 Ribosomal protein S6 kinase (p70S6K), HIF-1α, and c-Myc were respectively targeted by the PI3K/mTOR pathway to modulate the glucose homeostasis, glycolytic enzyme and OXPHOS [40–42]. Being consistent with the upregulation of PI3K/AKT/mTOR pathway, levels of p70S6K phosphorylation and expression of HIF-1α and c-Myc in MscL-G22S-activated SCs were also significantly higher than those in the other controls (Fig. 8D-E). When compared to SCs without stretching, the stretched SCs also activated AKT pathway and upregulation of HIF-1α and c-Myc (Fig. 8A-E).

To investigate the roles of PI3K/AKT/mTOR pathway in the action mechanism of MscL-G22S activation on energy metabolism of SCs, a potent inhibitor of PI3K, LY294002, was used. We found that the cell viability of SCs increased by MscL-G22S activation was significantly abolished, although the Ca^2+^ influx was not affected (Fig. 8F-G). The MscL-G22S-activated SCs with PI3K inhibitor had significantly less protein expression of HIF-1α and c-Myc and mRNA expression of GLUT1, GLUT4, HK2, PFKFB3, PKM2 and LDHA than the control SCs (Fig. 8H-I and Fig. S9A). Furthermore, the levels of glucose uptake and the intracellular glucose-6-phosphate, pyruvate, and lactate were also reduced by inhibition of PI3K (Fig. 8J-M). For the OXPHOS, the increased intracellular levels of NAD^+^ and the ratio of NAD^+^/NADH in MscL-G22S-activated SCs were also significantly reduced upon inhibition of PI3K (Fig. 8N-O).

**Fig. 9:**
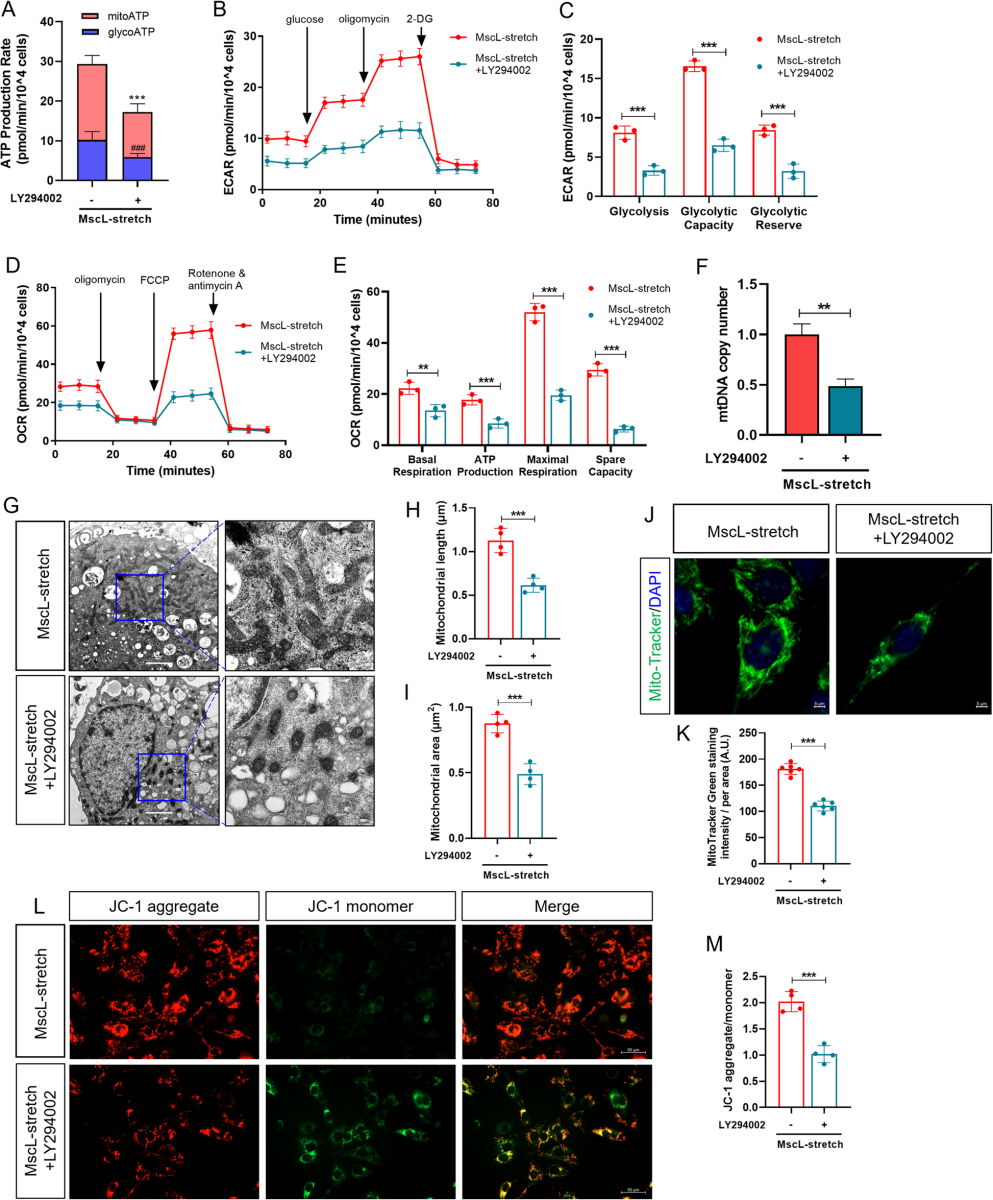
Inhibition of PI3K downregulated both glycolysis and OXPHOS in MscL-G22S-activated SCs. MscL-G22S-expressing SCs were pretreated with 10 mM of LY294002 and then mechanically stretched for 24 h. **A** The real-time ATP rate assay showed a significant reduction of mitochondrial and glycolytic ATP production in MscL-G22S-activated SCs by inhibition of PI3K. ***p < 0.001, mitochondrial ATP production; ^###^*p* < 0.001, glycolytic ATP production. (B-E). The Seahorse experiments showed that in MscL-G22S-activated SCs, inhibition of PI3K significantly abolished the upregulation of glycolysis rate, glycolytic capacity and glycolytic reserve in ECAR (**B**-**C**) and the basal respiration, ATP production, maximal respiration, and spare respiratory capacity in OCR (**D**-**E**). **F**-**K** The mtDNA copy number, mitochondrial length and area as well as the fluorescence intensity of mitochondria in MscL-G22S-activated SCs were significantly reduced by inhibition of PI3K. Scale bars = 2 μm for electron microscope and scale bars = 5 μm for mitochondria staining. **L**-**M** Analysis of mitochondrial membrane potential in MscL-G22S-activated SCs measured by JC-1 staining showed significantly less aggregated JC-1, indicating reduced mitochondrial activity caused by inhibition of PI3K. Scale bars = 50 μm. The data shown were representative of three or six independent experiments. NC, negative control; MscL, MscL-G22S-expressing SCs; NC-stretch, negative control with mechanical stretching; MscL-stretch, MscL-G22S-expressing SCs with mechanical stretching. **p < 0.01, ***p < 0.001

We further analyzed ATP production rate, ECAR and OCR of MscL-G22S-activated SCs upon PI3K inhibitor treatment using Seahorse examination. In comparison to the control, glycolytic ATP production rate, mitochondrial ATP production rate, ECAR and OCR were significant decreased upon LY294002 treatment in MscL-G22S-activated SCs (Fig. 9A-E). In detail, the glycolysis rate, glycolytic capacity, glycolytic reserve correlated with ECAR and the basal respiration, ATP production, maximal respiration and respiratory capacity correlated with OCR were remarkedly decreased in MscL-G22S-activated SCs upon PI3K inhibition (Fig. 9C, E), which is consistent with the reduction of glucose uptake, glycolytic metabolites, lactate production, and NAD^+^ content as shown above.

We then further investigated the effects of PI3K inhibition on mtDNA copy numbers and gene expression of mitochondrial ETC complexes. The treatment of LY294002 significantly decreased the mtDNA copy number and downregulated the transcriptional levels of mt-ND2, SDHA, mt-Cytb, mt-CO1 and mt-Atp8 in MscL-G22S-activated SCs (Fig. 9F and Fig. S9B). In addition, the length, area and number of mitochondria were significantly reduced upon the treatment of LY294002 in MscL-G22S-activated SCs (Fig. 9G-K). In line with changes in mitochondrial morphology and number, MMP indicated by JC-1 fluorescence was also reduced (Fig. 9L-M), suggesting inhibition of mitochondrial function in MscL-G22S-activated SCs by LY294002. Taken together, our data demonstrate that PI3K and its downstream of HIF-1α and c-Myc act as the core role in the action mechanism of MscL-G22S activation by mechanical stretching on upregulation of glycolysis, OXPHOS, and ATP production.

## Discussion

Adaptive transformation of energy metabolism patterns empowers the SCs with the ability to maintain the functional plasticity of peripheral nerve and facilitate structural repair after injury [8]. During the process of nerve regeneration, there is a significant increase in glycolysis within SCs. This upregulation serves multiple purposes, including facilitating rapid energy production for SC proliferation, clearance of nerve debris, and remyelination [43, 44]. Additionally, it provides an abundance of energetic substrates that contribute to axon regeneration through glial-axonal metabolic coupling [45, 46]. Physical exercise has been shown to potentiate the SCs proliferation and enhance axonal outgrowth [47, 48]; however, its action mechanism remains not clear. In this study, we demonstrated that mechanical stretching, the direct impact of exercise training on peripheral nerves, significantly promoted the energy metabolism of SCs by activating MscL-G22S. Because high-intensity mechanical stretching (such as > 20%) often results in cell damage [49], we hereby selected low-intensity stretching within the physiological range (usually < 10%) as the mechanical stimulus for the cultured SCs. Typically, engineered expression of MscL-G22S in SCs activated by low-intensity mechanical stretching induces a significant increase of Ca^2+^ influx and upregulation of glycolytic flux, oxidative metabolism, and lipid synthesis in cultured SCs with significant changes in a series of metabolic-related signaling and energic substrates, which may be translationally applied for the nerve regeneration.

After sensing mechanical stretching, MscL opens to form a pore with a diameter > 30 Å, which directly yields the diffusion and transport of large organic ions and small proteins inside and outside the SCs. We observed a significantly stretch-evoked increase of Ca^2+^ influx into the cultured SCs, which may further lead to the Ca^2+^ influx into mitochondria and nuclei. Mitochondrial Ca^2+^ homeostasis is associated with critical physiological functions including regulation of mitochondrial metabolism and ATP production while the nuclei Ca^2+^ influx affects gene transcription and cell viability [50, 51]. As expected, being along with the increase of Ca^2+^ influx, the viability of SCs with MscL-G22S activation was significantly promoted. Typically, intracellular Ca^2+^ is transiently elevated upon activation of mechanosensitive channels by a single stimulus and will then return to the basal level [19, 26]. However, continuous stimuli can activate the mechanosensitive channels to induce a persistent increase of intracellular Ca^2+^ for a period of time [26, 52]. Our study demonstrated that MscL-G22S activation is able to induce the persistent Ca^2+^ influx into the Schwann cells within a period of continuous mechanical stretching, which may be abolished by a self-protection mechanism of the cells when a certain threshold of intracellular Ca^2+^ concentration is exceeded.

The benefits of SCs from MscL-G22S activation prompted us to further investigate the potential mechanism. The proteomic analysis of SCs with different treatments displayed a variety of DEPs associated with the cellular process and signaling transduction. For the MscL-G22S-activated SCs and mechanical stretching alone SCs, the GO analysis showed that the DEPs were mainly associated with phosphorylation, regulation of signaling transduction, cell motility and migration, which are consistent with the significantly enhanced phosphorylation of PI3K/AKT/mTOR signaling pathway and reflect the status of SCs under mechanical stretching. The KEGG analysis showed the most of the DEPs between MscL-G22S-activated SCs and mechanical stretching alone SCs were associated with cancer, reactive oxygen species and AMP-activated protein kinase signaling pathway. It is not clear that why these DEPs were associated with cancer, which may be explained that both the MscL-G22S-activated SCs and the cancer cells may share similar characters of high levels of energy metabolism. For the comparison between MscL-G22S-activated SCs and MscL-G22S expressing alone SCs or the control SCs without treatment, both the GO and KEGG analysis showed the DEPs were mainly associated with the metabolic and biosynthetic process, which is also observed in the analysis of DEPs between the control SCs and those with stretching only. These findings together with the data of energy metabolomics analysis both supported the significant upregulation of energy metabolism in SCs caused by MscL-G22S activation. Notably, GO analysis showed that a majority of DEPs between SCs with or without mechanical stretching located in the nucleus, indicating significant transcriptional changes caused by mechanical stimulus. Furthermore, most DEPs between MscL-G22S-activated SCs and those with mechanical stretching alone located in the plasma membrane, which may contribute to the regulation of sensing of mechanical stimulus and transmembrane signaling receptor activity, associated with the intracellular and extracellular substrate transport.

Both the proteomic and targeted energy metabolomics analysis indicated energy metabolism as the main action mechanism of MscL-G22S-activation on SCs. We then further investigated the details of changes in energy metabolism of the SCs with different treatments. In the nervous system, glial cells actively uptake glycose, produce the energic substrates via glycolysis, for example, the lactate, and then provide the energy supply for the neuron and the axons through a coupling mechanism [53, 54]. As the class I transporters, GLUT1 and GLUT4 are the most extensively studied GLUTs in mammalian tissues. Dramatic upregulation of GLUT1 and GLUT4 expression promotes glial cell activation by facilitating glycolysis [34, 54]. In our study, under the mechanical stimuli, MscL-G22S activation significantly upregulated the GLUT1 and GLUT4 expression in SCs, which indicates the escalated glucose absorption and glycolysis initiation. Furthermore, in the MscL-G22S-activated SCs, we observed augmented expression of the key glycolytic enzymes, HK2, PKM2, and LDHA, along with the upregulated PFKFB3, indicating heightened glycolytic activity. The targeted energy metabolomics analysis indicated that in the MscL-G22S-activated SCs, the accumulated pyruvate and lactate may be further converted to acetyl-CoA within the TCA cycle, which can be consumed in the amino acid metabolism, lipid synthesis, and oxidative pathway. The increased NAD^+^ content, paired with the upregulated expression of mitochondrial ETC complexes and higher levels of MMP in MscL-G22S-activated SCs demonstrated the significant enhancement of OXPHOS. Our data of mitochondrial analysis including the mtDNA copy number, number, length and area of mitochondria as well as the TFAM expression demonstrated the significant upregulation of mitochondrial activity induced by MscL-G22S activation in cultured SCs, which permits a remarkable mitochondrial respiratory and OXPHOS. Additionally, energy metabolomics analysis showed a remarkable surge in glycolytic products, nucleotide, and amino acid products, indicating that mechanical stretching-induced MscL-G22S activation causes significantly upregulated levels of glycolysis, OXPHOS, nucleotide metabolism, amino acids metabolism, and lipid metabolism.

The nutrient availability and energy demand usually determine the rate of glycolysis relative to oxidative metabolism in specific cells. After nerve injury, a rapid glycolytic shift in SCs favors the survival of axons [8, 53–55]. Unexpectedly, in the cultured MscL-G22S-expressing SCs subjected to mechanical stretching, we observed simultaneous enhancement of glycolysis and OXPHOS, indicated by increased ECAR, OCR, and glycolytic and mitochondrial ATP production rates. The mechanism of simultaneous enhancement of glycolysis and OXPHOS is not clear. It is possible that in the cultured conditions, in the absence of a glial-axon coupling mechanism, the massive production of glycolysis substrates will be further consumed by OXPHOS to prevent the abnormal accumulation of these substrates. A further study using a neuron-Schwann cells coculture system is necessary to confirm our hypothesis. Notably, we observed that mechanical stretching alone also induces a slight increase of Ca^2+^ influx and cell activity of cultured SCs, which has been reported by other researchers [56, 57]. Some endogenously expressed mechanosensitive channels, for example, the Piezo channels [19], may sense the mechanical stimulus on the cell membrane and transform the stretching effects into the signaling pathway of promoting the Ca^2+^ influx and cell activity of cultured SCs. Additionally, when compared to the MscL-G22S activation, mechanical stretching alone exerts slight effects on the upregulation of glycolysis and OXPHOS as well as several mitochondrial ETC complexes; however, mechanical stretching alone didn’t impact mitochondrial dimensions in the SCs. The positive and mild modulation of mechanical stretching alone on the energy metabolism of SCs may aid the glial-axonal metabolic coupling and promote nerve regeneration, which may partly explain the mechanism of therapeutic effects of physical exercise on nerve injury with low efficiency. Furthermore, under mechanical stretching, the mild upregulation of viability and energy metabolism in SCs induced by activation of endogenous mechanosensitive channels may promote the interaction between SCs and nociceptive neurons, which may be applied to explain the mechanism of the relief of neuropathic pain following stretching [58–60]. Given that the MscL-G22S sensing mechanical stretching induced more significant upregulation of energy metabolism of cultured SCs than mechanical stretching alone, recruiting SCs with engineered expression of MscL-G22S into the impaired site of peripheral nerve and then adding the exogenous mechanical stimulus may serve as a potential strategy for peripheral nerve impairment.

We further investigated the key factors in the energy metabolic signaling pathway of SCs modulated by MscL-G22S activation. As a highly conserved intracellular signaling pathway, the PI3K/AKT/mTOR cascade critically mediates the transduction of extracellular stimuli into the cellular response, including a series of phospholipid and protein phosphorylation of various downstream substrates, which orchestrates the cell growth, survival, and metabolism [40, 61, 62]. Upregulation of PI3K/AKT/mTOR promoting axonal growth and regeneration has been applied in the mechanism of therapeutic strategies for nerve injury [63–65]. As a serine/threonine kinase regulated by PI3K/mTOR, p70S6K critically participates in the modulation of the cell cycle, growth, and survival. Upon activation, p70S6K phosphorylates the S6 protein of ribosomal subunit 40S leading to the selective translation of protein synthesis-related mRNA family [66–68]. In this study, we observed that MscL-G22S activation but not the mechanical stretching alone induced significant phosphorylation of PI3K/AKT/mTOR signaling and upregulated expression of the downstream targets, HIF-1α and c-Myc, as well as higher levels of p70S6K phosphorylation. In addition to activating GLUT1 and LDHA, HIF-1α and c-Myc cooperate to promote the expression of HK2 and pyruvate dehydrogenase kinase 1, resulting in increased conversion of glucose to lactate, which finally contributed to the Warburg effect [69]. HIF-1α and c-Myc also synergistically induce the expression of regulators of amino acid, nucleotide, and lipid synthesis [8, 70, 71]. Together with previous findings, our study explained the action mechanism of mechanical stretching stimulating the MscL-G22S on the energy metabolism of SCs. We also demonstrated that inhibition of PI3K activation by its inhibitor, LY294002, significantly abolished the upregulation of glycolysis and OXPHOS by MscL-G22S activation in SCs, leading to a decrease of glucose uptake and production of energic substrates including pyruvate, lactate, NAD^+^, and ATP, etc. Additionally, inhibition of PI3K caused the reduction of HIF-1α and c-Myc expression in MscL- G22S-activated SCs. Our data demonstrated a central role of PI3K in the action mechanism of MscL-G22S activation-induced upregulation of the energy metabolism of SCs and confirmed HIF-1α/c-Myc as the downstream targets of the PI3K signaling pathway.

**Fig. 10:**
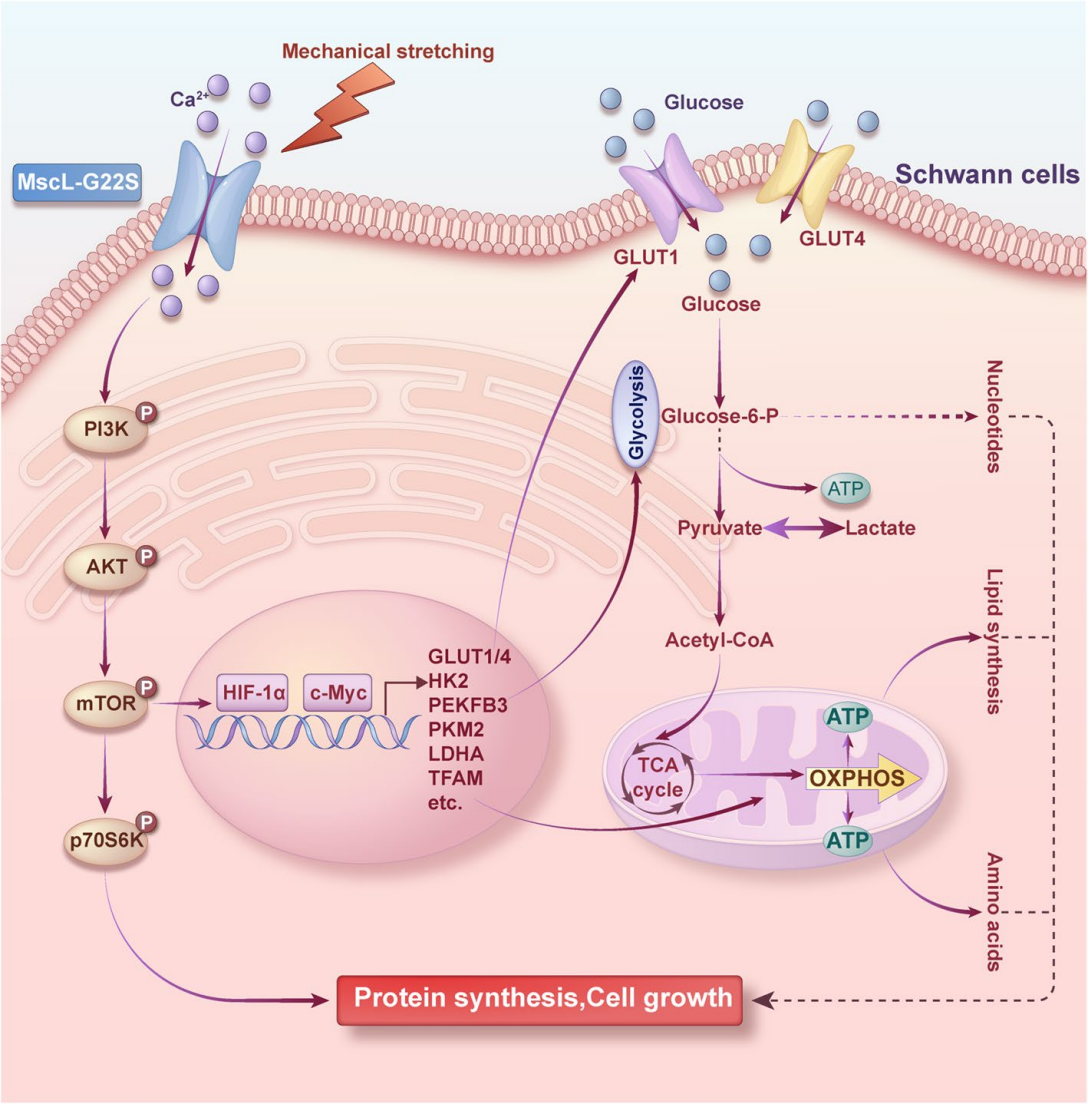
MscL-G22S activation by mechanical stretching promotes Ca^2+^ influx and increased energy metabolism in cultured Schwann cells through the PI3K/AKT/mTOR pathway and its downstream HIF-1α/c-Myc signaling (by Figdraw)

In conclusion, the study demonstrates that mechanical stretching-activated MscL-G22S caused significant upregulation of energy metabolism in SCs, including both glycolysis and OXPHOS with a series of changes in metabolomics and proteomic profiles, especially the PI3K/AKT/mTOR pathway and its downstream HIF-1α/c-Myc signaling (Fig. 10). Mechanical stretching alone also induced mild increases of cell viability and energy metabolism in SCs by activating glycolysis and OXPHOS. Our findings may be helpful in explaining the mechanism of therapeutic effects of physical exercise on nerve regeneration and relief of neuropathic pain. Combination therapy utilizing the spatiotemporally engineered expression of MscL-G22S on SCs in peripheral nerve and exogenous mechanical stimulation may increase the energic supports for the axonal regeneration and nerve repair, which may be a potential strategy for the clinical treatment of peripheral neuropathy and deserve to be validated using the animal model.

### Supplementary Information

**Additional file 1: Supplemental Table** **1.** Primer sequences for RT-qPCR. **Supplemental Table 2**. Details of primary and secondary antibodies.

**Additional file 2: Supplemental Fig. 1.** The plasmid map of pLentai-hEF1a-PuroR-CMV-MscL-G22S-mCherry.

**Additional file 3: Supplemental Fig. 2.** Proteomic analysis of mechanical stretching and MscL-G22S activation in SCs. (A). PCA plot shows separation between different groups and repeatability of intra group samples. (B). Subcellular localization of DEPs from different groups. (B). Volcano plot of DEPs from different groups. GO: cellular component (C) and molecular function (D) enrichment analysis based on DEPs from different groups. PCA: Principal-component analysis. NC, negative control; MscL, MscL-G22S-expressing SCs; NC-stretch, negative control with mechanical stretching; MscL-stretch, MscL-G22S-expressing SCs with mechanical stretching.

**Additional file 4: Supplemental Fig. 3**. Energy metabolite analysis between NC group and NC-stretch group. (A). Quantification of differential metabolites. (B). Summary of metabolic pathways and targets affected by mechanical stretching in SCs. A schematic diagram is made to show the changes in metabiotic pathways of glycolysis, TCA cycle, OXPHOS and purine/pyrimidine metabolism between NC group and NC-stretch group. Significantly higher metabolites in NC-stretch group compared to NC group were indicated in red. NC, negative control; NC-stretch, negative control with mechanical stretching.

**Additional file 5: Supplemental Fig. 4.** Energy metabolite analysis between NC group and MscL-G22S-stretch group. (A). Quantification of differential metabolites. (B). Summary of metabolic pathways and targets affected by mechanical stretching in SCs. A schematic diagram is made to show the changes in metabiotic pathways of glycolysis, TCA cycle, OXPHOS and purine/pyrimidine metabolism between NC group and MscL-G22S-stretch group. Significantly higher and lower metabolites in MscL-stretch group compared to NC group were indicated in red and green, respectively. NC, negative control; MscL-stretch, MscL-G22S-expressing SCs with mechanical stretching.

**Additional file 6: Supplemental Fig. 5.** Energy metabolite analysis between MscL-G22S group and MscL-G22S-stretch group. (A). Quantification of differential metabolites. (B). Summary of metabolic pathways and targets affected by mechanical stretching in SCs. A schematic diagram is made to show the changes in metabiotic pathways of glycolysis, TCA cycle, OXPHOS and purine/pyrimidine metabolism between MscL-G22S group and MscL-G22S-stretch group. Significantly higher and lower metabolites in MscL-G22S-stretch group compared to NC group were indicated in red and green, respectively. NC, negative control; MscL-stretch, MscL-G22S-expressing SCs with mechanical stretching.

**Additional file 7: Supplemental Fig. 6.** Energy metabolite analysis between NC-stretch group and MscL-G22S-stretch group. (A). Quantification of differential metabolites. (B). Summary of metabolic pathways and targets affected by mechanical stretching in SCs. Shown is a schematic representation of metabiotic pathways of glycolysis, TCA cycle, OXPHOS and purine/pyrimidine metabolism. Significantly higher and lower metabolites in MscL-stretch group compared to NC-stretch group were indicated in red and green, respectively. NC-stretch, negative control with mechanical stretching; MscL-stretch, MscL-G22S-expressing SCs with mechanical stretching.

**Additional file 8: Supplemental Fig. 7.** Mechanical stretching upregulated the expression levels of glycolysis-related proteins in MscL-G22S-activated SCs. (A). Representative Immunofluorescence of GLUT4, PKM2 and LDHA in SCs. (B). Quantification of the fluorescence intensity of GLUT1, GLUT4, HK2, PFKFB3, PKM2, and LDHA in SCs. The data shown were representative from four independent experiments. NC, negative control; MscL, MscL-G22S-expressing SCs; NC-stretch, negative control with mechanical stretching; MscL-stretch, MscL-G22S-expressing SCs with mechanical stretching. *ns*, not significant, *p < 0.05, **p < 0.01, ***p < 0.001.

**Additional file 9: Supplemental Fig. 8. **Mechanical stretching up-regulated the expression levels of mitochondrial electron transfer chain proteins and TFAM in MscL-G22S-expressing SCs. (A). Representative Immunofluorescence of SDHA and mt-Cytb in SCs. (B). Quantification of the fluorescence intensity of mitochondrial electron transfer chain proteins and TFAM in SCs. The data shown were representative from four independent experiments. NC, negative control; MscL, MscL-G22S-expressing SCs; NC-stretch, negative control with mechanical stretching; MscL-stretch, MscL-G22S-expressing SCs with mechanical stretching. *ns*, not significant, *p < 0.05, **p < 0.01, ***p < 0.001.

**Additional file 10: Supplemental Fig. 9.** Inhibition of PI3K reduced the expression of glycolytic and mitochondrial ETC component genes in MscL-G22S-activated SCs. (A-B). The glycolysis-related mRNAs (A) and mitochondrial ETC components (B) in MscL-G22S-activated SCs were all significantly reduced by inhibition of PI3K. The data shown were representative from four independent experiments. MscL-stretch, MscL-G22S-expressing SCs with mechanical stretching. *p < 0.05, **p < 0.01, ***p < 0.001.

## Data Availability

The data are available from the corresponding author on reasonable request.
