# Supplementary material for: Mechanosensitive channel of large conductance enhances the mechanical stretching-induced upregulation of glycolysis and oxidative metabolism in Schwann cells
Source: Cell Commun Signal. 2024 Feb 1;22:93. doi: 10.1186/s12964-024-01497-x (PMC10835878; doi:10.1186/s12964-024-01497-x)
Supplement: Supplementary file 1 — Additional file 1: Supplemental Table 1. Primer sequences for RT-qPCR. Supplemental Table 2. Details of primary and secondary antibodies. [file 12964_2024_1497_MOESM1_ESM.docx]

**Supplemental materials**

**Supplemental Table 1. Primer sequences for RT-qPCR.**

| Gene |  | Primer sequence (5’-3’) |
| --- | --- | --- |
| MscL | Forward | GTCTCTTCACTGGTTGCCGA |
|  | Reverse | TGCATCACAACAGCAGGGAT |
| GLUT1 | Forward | CAGTTCGGCTATAACACTGGTG |
|  | Reverse | GCCCCCGACAGAGAAGATG |
| GLUT4 | Forward | GGACCGGATTCCATCCCAC |
|  | Reverse | TCCCAACCATTGAGAAATGATGC |
| PFKFB3 | Forward | CAACTCCCCAACCGTGATTGT |
|  | Reverse | GAGGTAGCGAGTCAGCTTCTT |
| PKM2 | Forward | GTGCCGCCTGGACATTGACTC |
|  | Reverse | ATTCAGCCGAGCCACATTCATCC |
| LDHA | Forward | TCGCACCTTGTAGCCGTTATTGG |
|  | Reverse | ACTGCCCTCCCGCTCTTCTC |
| Subunit 2 of cytochrome c oxidase | Forward | ATAACCGAGTCGTTCTGCCAAT |
|  | Reverse | TTTCAGAGCATTGGCCATAGAA |
| Rsp18 | Forward | GTGTTAGGGGACTGGTGGACA |
|  | Reverse | CATCACCCACTTACCCCCAAA |
| mt-ND2 | Forward | ACCAAATCTCTCCCTCACTAAACG |
|  | Reverse | CCACCTCAACTGCCTGCTATG |
| SDHA | Forward | TTACAAAGTGCGGGTCGATGA |
|  | Reverse | TGTTCCCCAAACGGCTTCTT |
| mt-Cytb | Forward | CCCACCCCATATTAAACCCG |
|  | Reverse | GAGGTATGAAGGAAAGGTATAAGGG |
| mt-CO1 | Forward | TCCCAGATATAGCATTCCCACG |
|  | Reverse | ACTGTTCATCCTGTTCCTGC |
| *mt-Atp8* | Forward | GCCACAACTAGATACATCAACATG |
|  | Reverse | TGGTTGTTAGTGATTTTGGTGAAG |
| GAPDH | Forward | AACGACCCCTTCATTGAC |
|  | Reverse | TCCACGACATACTCAGCAC |

**Supplemental table 2. Details of primary and secondary antibodies**

| Antibodies | Cat. number | Applications | Source |
| --- | --- | --- | --- |
| Mouse anti-mCherry-Tag | AE002 | WB: 1:5000 | ABclonal |
| Rabbit anti-GLUT1 | ab115730 | WB: 1:500 IF: 1:200 | Abcam |
| Mouse anti-GLUT4 | BF1001 | WB: 1:500 IF: 1:200 | Affinity |
| Rabbit anti-PFKFB3 | ab181861 | WB: 1:1000 IF: 1:200 | Abcam |
| Rabbit anti-LDHA | DF6280 | WB: 1:1000 IF: 1:200 | Affinity |
| Rabbit anti-HK2 | DF6176 | WB: 1:2000 IF: 1:400 | Affinity |
| Rabbit anti-PKM2 | AF5234 | WB: 1:1000 IF: 1:200 | Affinity |
| Rabbit anti-mt-ND2 | A17968 | WB: 1:500 IF: 1:100 | ABclonal |
| Rabbit anti-SDHA | 14865-1-AP | WB: 1:1000 IF: 1:200 | Proteintech |
| Rabbit anti-mt-Cytb | 55090-1-AP | WB: 1:1000 IF: 1:200 | Proteintech |
| Rabbit anti-mt-CO1 | A7341 | WB: 1:500 IF: 1:100 | ABclonal |
| Rabbit anti-mt-ATP8 | 26723-1-AP | WB: 1:2000 IF: 1:400 | Proteintech |
| Rabbit anti-PI3K p85α | AF6241 | WB: 1:1000 | Affinity |
| Rabbit anti-p-PI3K p85α (Tyr607) | AF3241 | WB: 1:500 | Affinity |
| Rabbit anti-AKT | AF6261 | WB: 1:1000 | Affinity |
| Rabbit anti-p-AKT (Ser473) | AF0016 | WB: 1:500 | Affinity |
| Rabbit anti-mTOR | AF6308 | WB: 1:1000 | Affinity |
| Rabbit anti-p-mTOR (Ser2448) | 5536 | WB: 1:1000 | Cell Signal Technology |
| Rabbit anti-p70S6K | AF6226 | WB: 1:1000 | Affinity |
| Rabbit anti-p-p70S6K (Thr389/Thr412) | AF3228 | WB: 1:500 | Affinity |
| Rabbit anti-HIF-1α | 20960-1-AP | WB: 1:2000 | Proteintech |
| Rabbit anti-c-Myc | 10828-1-AP | WB: 1:2000 | Proteintech |
| Mouse anti-GAPDH | AC033 | WB: 1:10000 | ABclonal |
| Goat anti-mouse IgG-HRP | AS064 | WB: 1:5000 | ABclonal |
| Goat anti-Rabbit IgG-HRP | AS063 | WB: 1:5000 | ABclonal |
| Goat anti-mouse IgG, Alexa Fluor™ 488 | A32723 | IF: 1:500 | ThermoFisher |
| Goat anti-rabbit IgG, Alexa Fluor™ 488 | A-11034 | IF: 1:500 | ThermoFisher |
