# Supplementary figures and images for: Mechanosensitive channel of large conductance enhances the mechanical stretching-induced upregulation of glycolysis and oxidative metabolism in Schwann cells
Source: Cell Commun Signal. 2024 Feb 1;22:93. doi: 10.1186/s12964-024-01497-x (PMC10835878; doi:10.1186/s12964-024-01497-x)

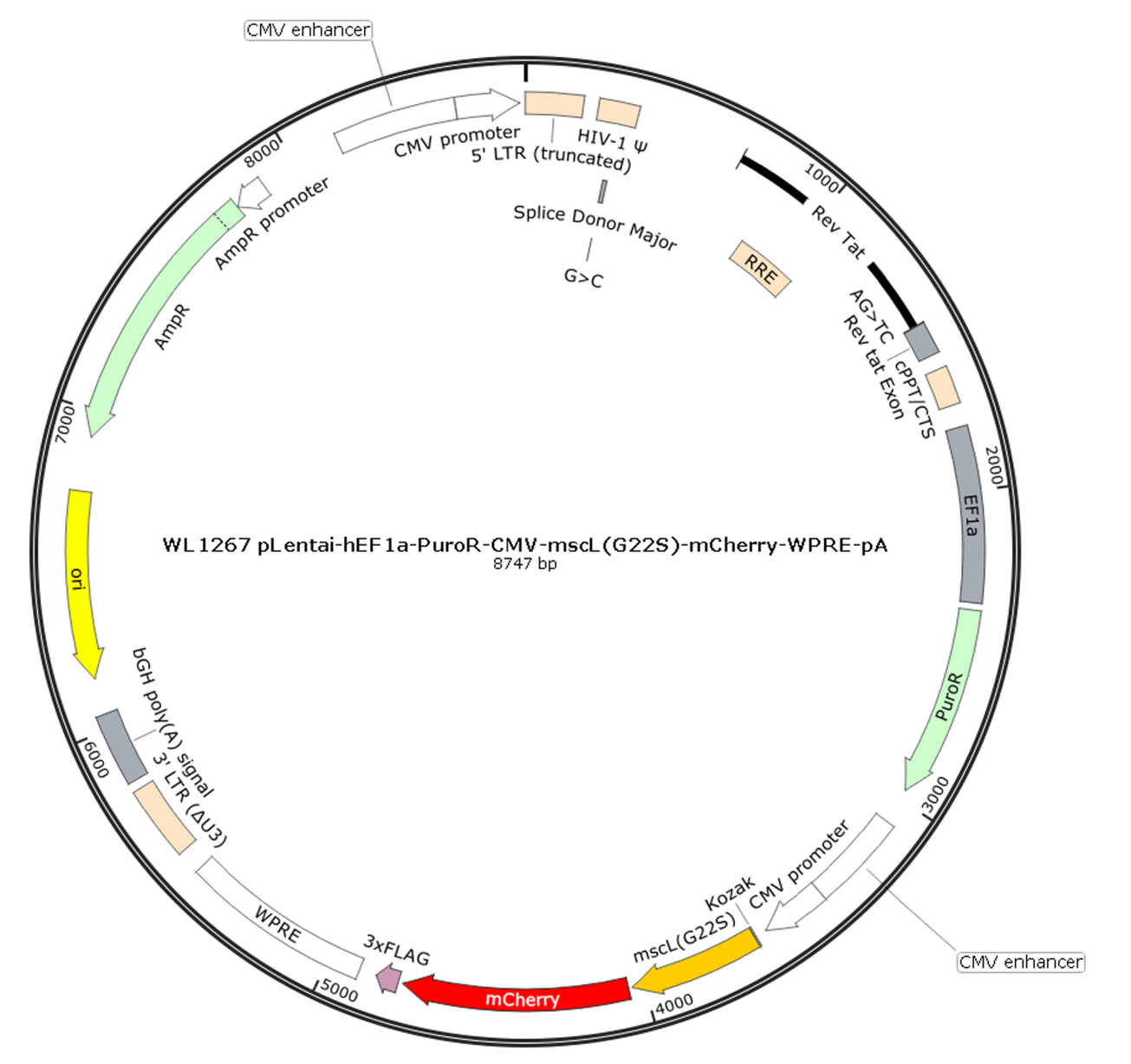

Supplement: Supplementary file 2 — Additional file 2: Supplemental Fig. 1. The plasmid map of pLentai-hEF1a-PuroR-CMV-MscL-G22S-mCherry. [file 12964_2024_1497_MOESM2_ESM.tif]

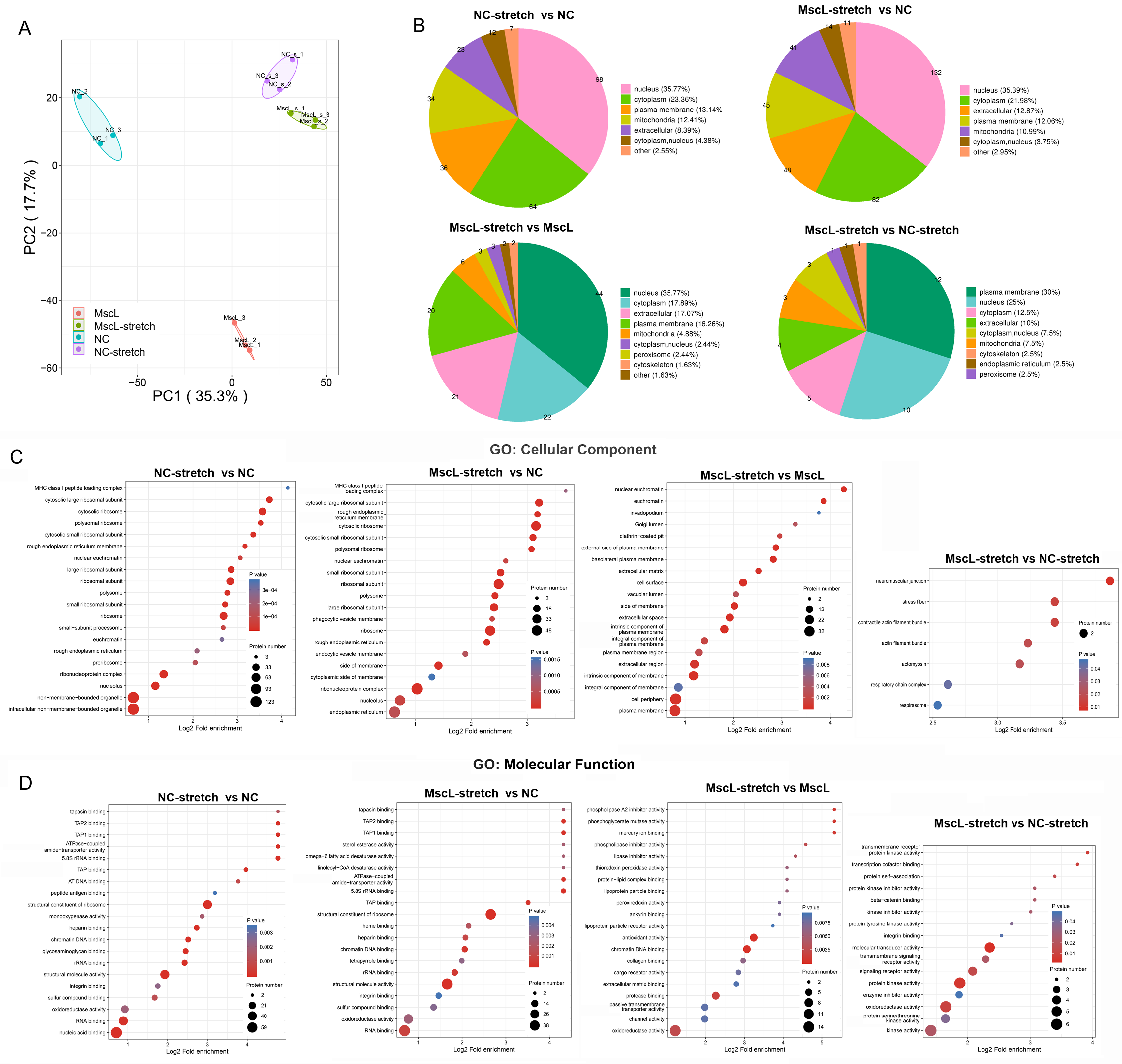

Supplement: Supplementary file 3 — Additional file 3: Supplemental Fig. 2. Proteomic analysis of mechanical stretching and MscL-G22S activation in SCs. (A). PCA plot shows separation between different groups and repeatability of intra group samples. (B). Subcellular localization of DEPs from different groups. (B). Volcano plot of DEPs from different groups. GO: cellular component (C) and molecular function (D) enrichment analysis based on DEPs from different groups. PCA: Principal-component analysis. NC, negative control; MscL, MscL-G22S-expressing SCs; NC-stretch, negative control with mechanical stretching; MscL-stretch, MscL-G22S-expressing SCs with mechanical stretching. [file 12964_2024_1497_MOESM3_ESM.tif]

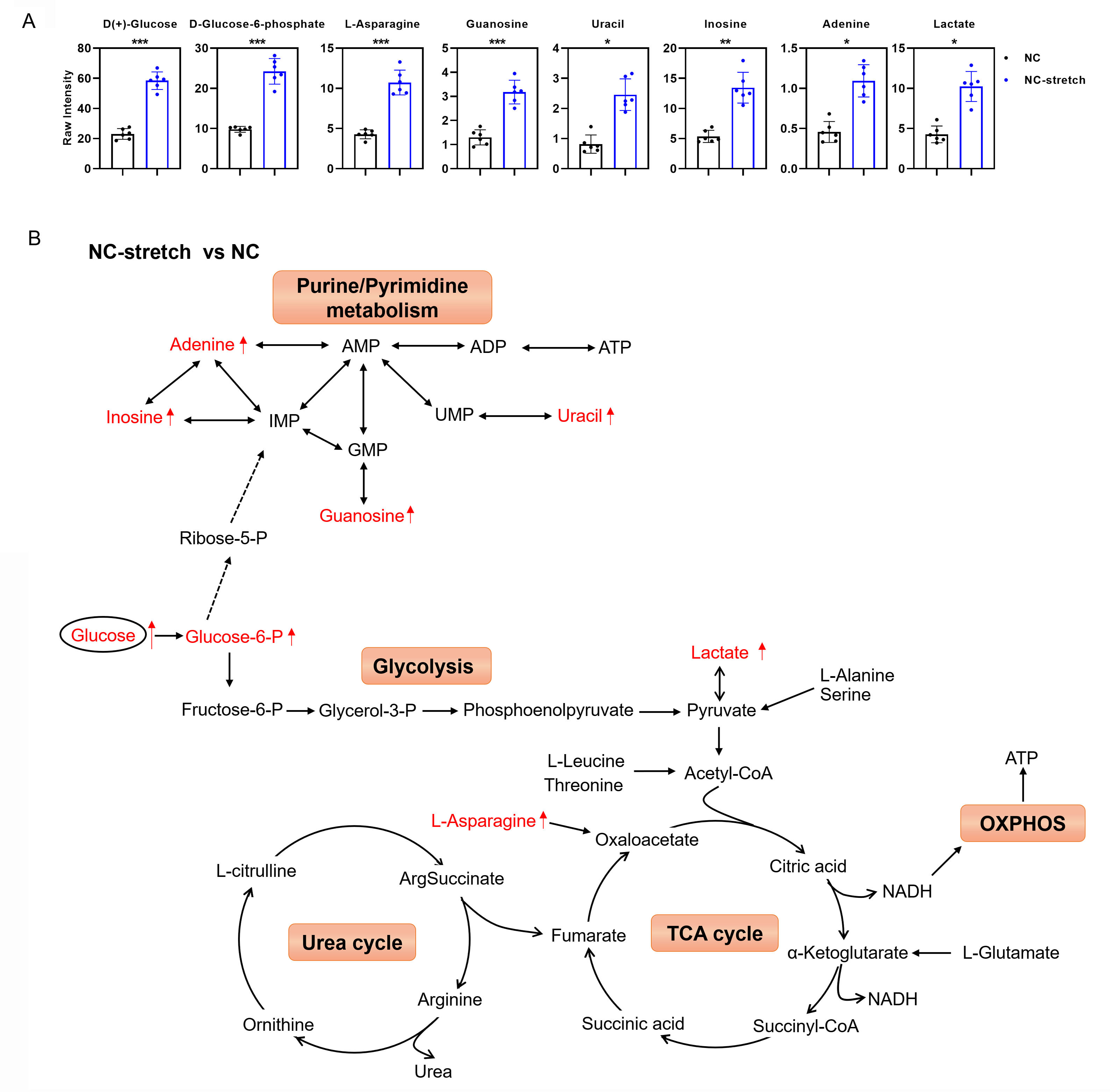

Supplement: Supplementary file 4 — Additional file 4: Supplemental Fig. 3. Energy metabolite analysis between NC group and NC-stretch group. (A). Quantification of differential metabolites. (B). Summary of metabolic pathways and targets affected by mechanical stretching in SCs. A schematic diagram is made to show the changes in metabiotic pathways of glycolysis, TCA cycle, OXPHOS and purine/pyrimidine metabolism between NC group and NC-stretch group. Significantly higher metabolites in NC-stretch group compared to NC group were indicated in red. NC, negative control; NC-stretch, negative control with mechanical stretching. [file 12964_2024_1497_MOESM4_ESM.tif]

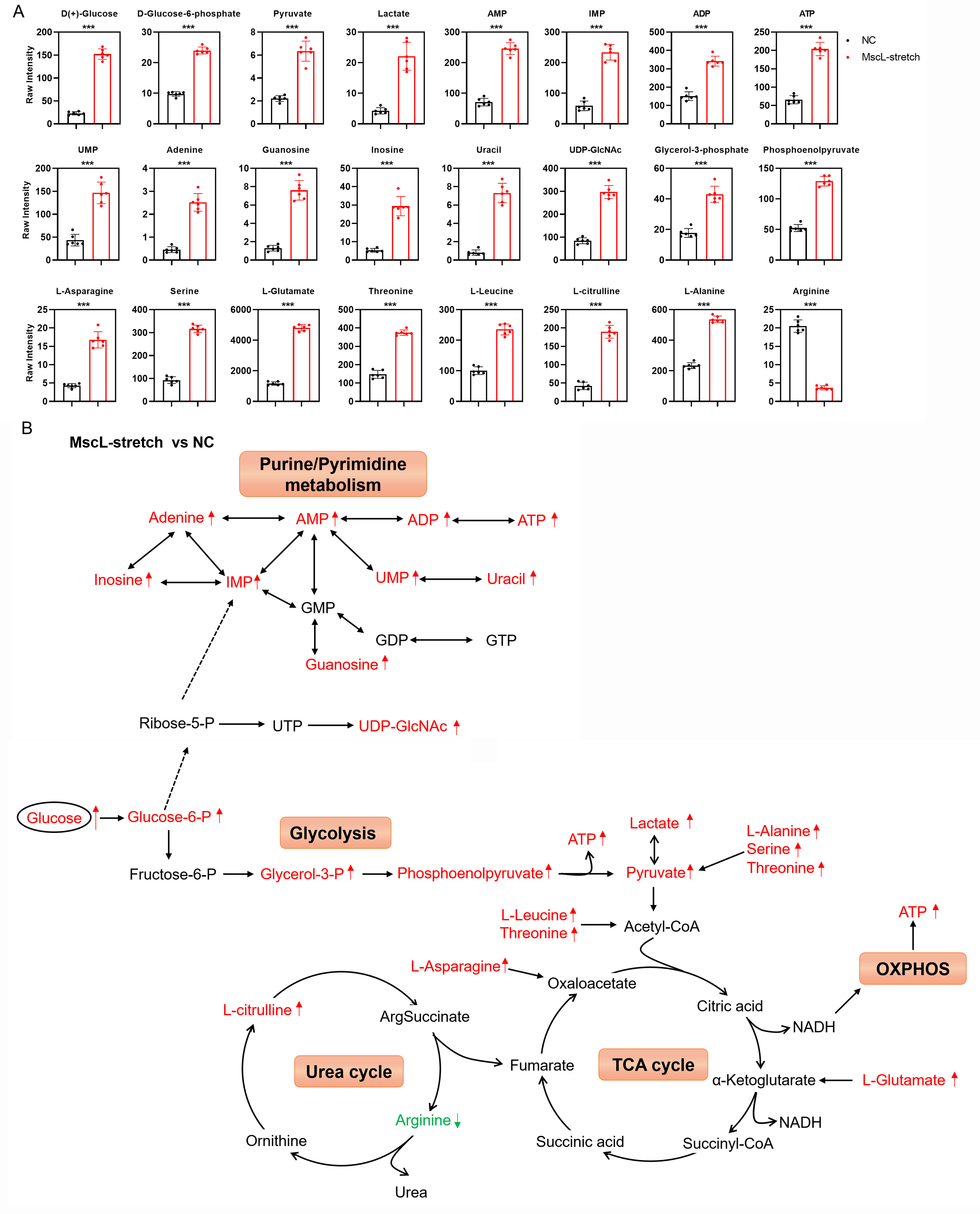

Supplement: Supplementary file 5 — Additional file 5: Supplemental Fig. 4. Energy metabolite analysis between NC group and MscL-G22S-stretch group. (A). Quantification of differential metabolites. (B). Summary of metabolic pathways and targets affected by mechanical stretching in SCs. A schematic diagram is made to show the changes in metabiotic pathways of glycolysis, TCA cycle, OXPHOS and purine/pyrimidine metabolism between NC group and MscL-G22S-stretch group. Significantly higher and lower metabolites in MscL-stretch group compared to NC group were indicated in red and green, respectively. NC, negative control; MscL-stretch, MscL-G22S-expressing SCs with mechanical stretching. [file 12964_2024_1497_MOESM5_ESM.tif]

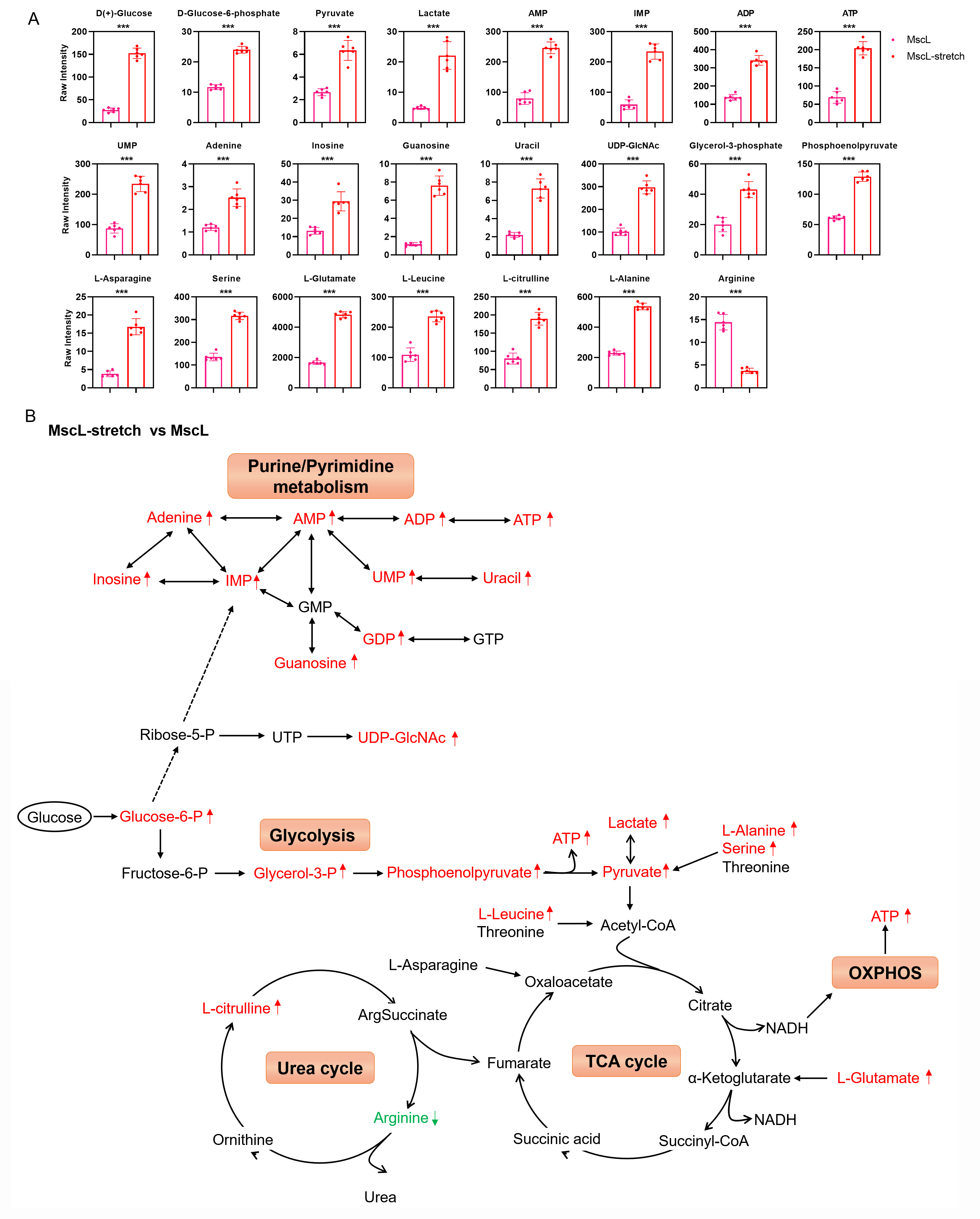

Supplement: Supplementary file 6 — Additional file 6: Supplemental Fig. 5. Energy metabolite analysis between MscL-G22S group and MscL-G22S-stretch group. (A). Quantification of differential metabolites. (B). Summary of metabolic pathways and targets affected by mechanical stretching in SCs. A schematic diagram is made to show the changes in metabiotic pathways of glycolysis, TCA cycle, OXPHOS and purine/pyrimidine metabolism between MscL-G22S group and MscL-G22S-stretch group. Significantly higher and lower metabolites in MscL-G22S-stretch group compared to NC group were indicated in red and green, respectively. NC, negative control; MscL-stretch, MscL-G22S-expressing SCs with mechanical stretching. [file 12964_2024_1497_MOESM6_ESM.tif]

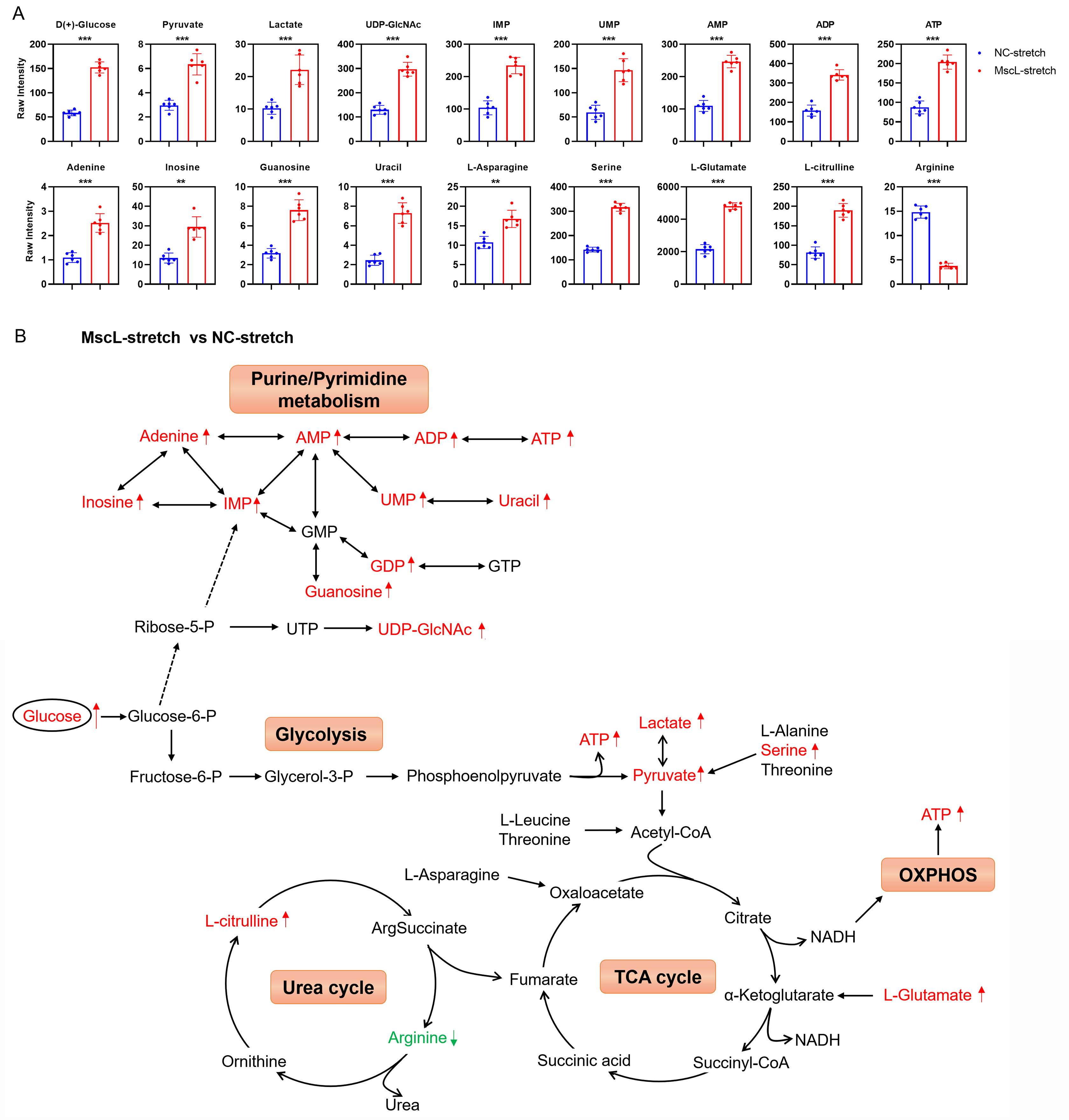

Supplement: Supplementary file 7 — Additional file 7: Supplemental Fig. 6. Energy metabolite analysis between NC-stretch group and MscL-G22S-stretch group. (A). Quantification of differential metabolites. (B). Summary of metabolic pathways and targets affected by mechanical stretching in SCs. Shown is a schematic representation of metabiotic pathways of glycolysis, TCA cycle, OXPHOS and purine/pyrimidine metabolism. Significantly higher and lower metabolites in MscL-stretch group compared to NC-stretch group were indicated in red and green, respectively. NC-stretch, negative control with mechanical stretching; MscL-stretch, MscL-G22S-expressing SCs with mechanical stretching. [file 12964_2024_1497_MOESM7_ESM.tif]

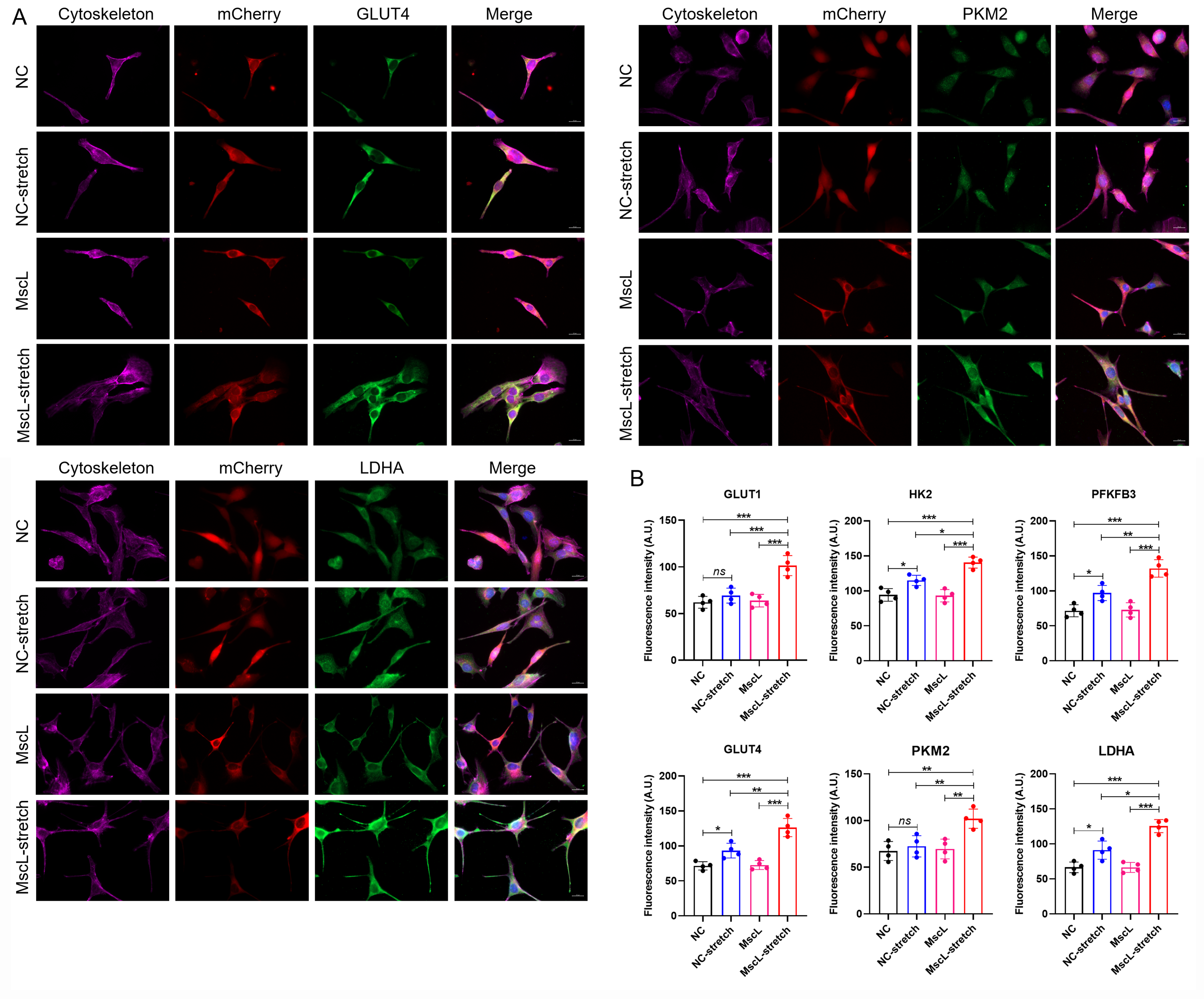

Supplement: Supplementary file 8 — Additional file 8: Supplemental Fig. 7. Mechanical stretching upregulated the expression levels of glycolysis-related proteins in MscL-G22S-activated SCs. (A). Representative Immunofluorescence of GLUT4, PKM2 and LDHA in SCs. (B). Quantification of the fluorescence intensity of GLUT1, GLUT4, HK2, PFKFB3, PKM2, and LDHA in SCs. The data shown were representative from four independent experiments. NC, negative control; MscL, MscL-G22S-expressing SCs; NC-stretch, negative control with mechanical stretching; MscL-stretch, MscL-G22S-expressing SCs with mechanical stretching. ns, not significant, *p < 0.05, **p < 0.01, ***p < 0.001. [file 12964_2024_1497_MOESM8_ESM.tif]

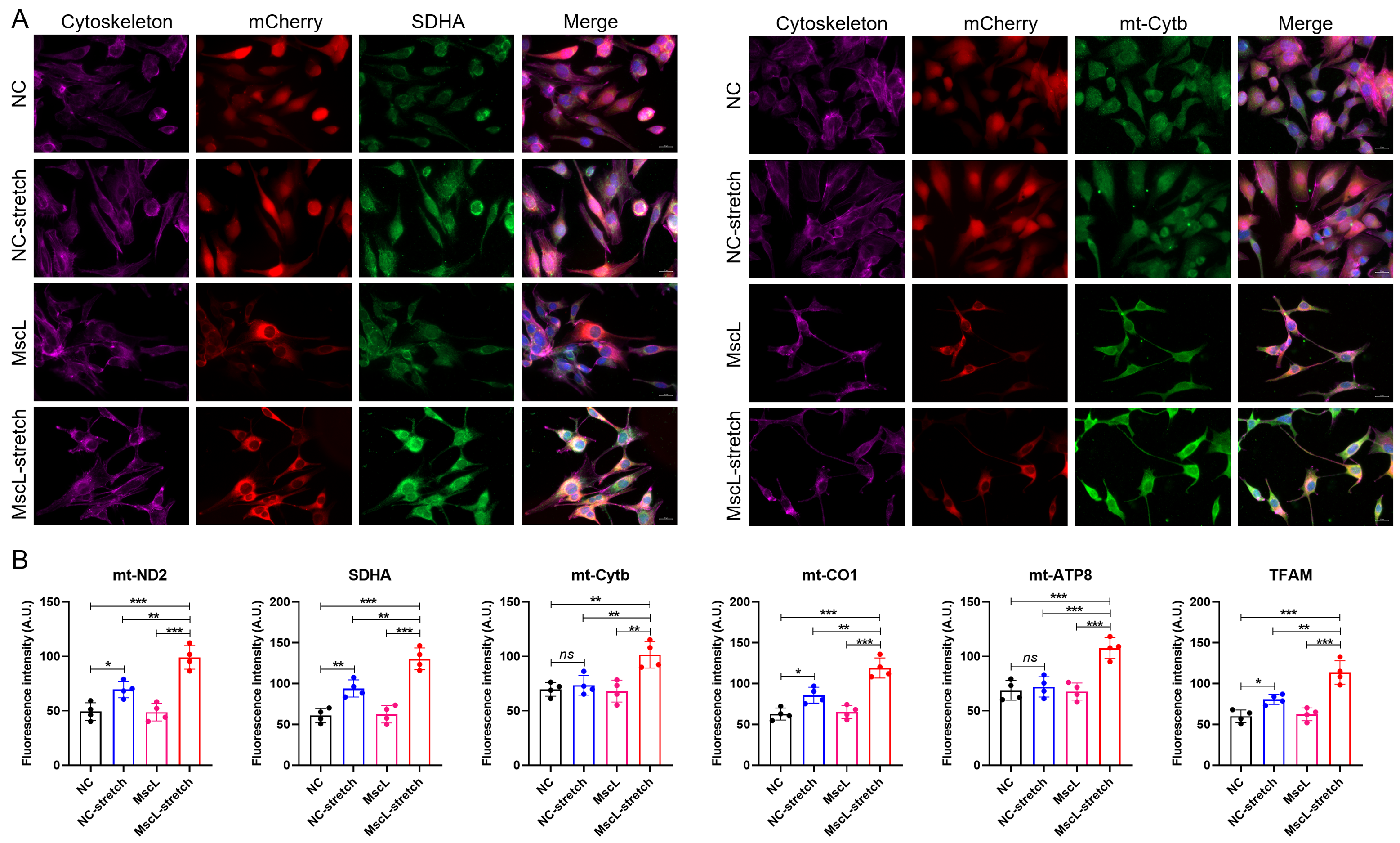

Supplement: Supplementary file 9 — Additional file 9: Supplemental Fig. 8. Mechanical stretching up-regulated the expression levels of mitochondrial electron transfer chain proteins and TFAM in MscL-G22S-expressing SCs. (A). Representative Immunofluorescence of SDHA and mt-Cytb in SCs. (B). Quantification of the fluorescence intensity of mitochondrial electron transfer chain proteins and TFAM in SCs. The data shown were representative from four independent experiments. NC, negative control; MscL, MscL-G22S-expressing SCs; NC-stretch, negative control with mechanical stretching; MscL-stretch, MscL-G22S-expressing SCs with mechanical stretching. ns, not significant, *p < 0.05, **p < 0.01, ***p < 0.001. [file 12964_2024_1497_MOESM9_ESM.tif]

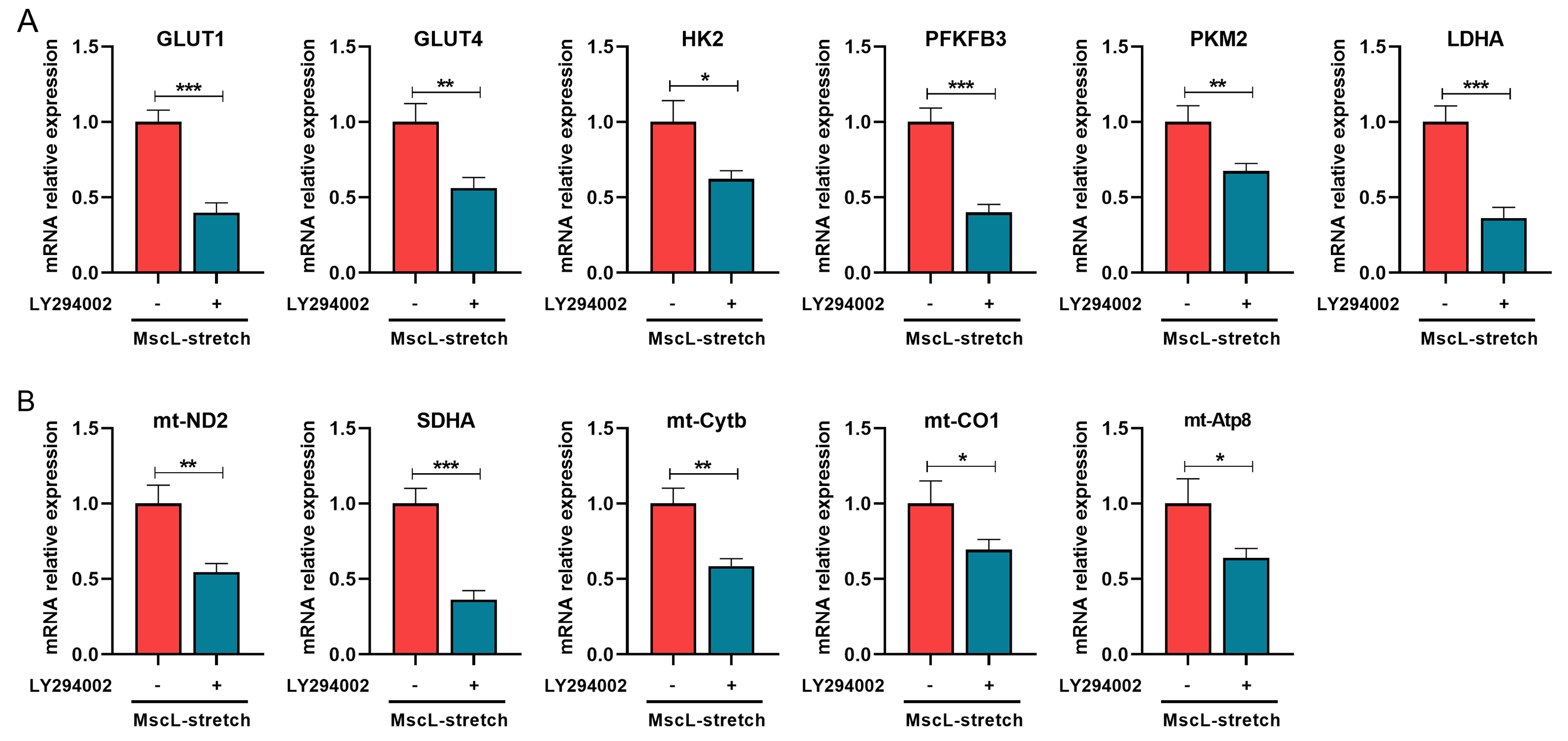

Supplement: Supplementary file 10 — Additional file 10: Supplemental Fig. 9. Inhibition of PI3K reduced the expression of glycolytic and mitochondrial ETC component genes in MscL-G22S-activated SCs. (A-B). The glycolysis-related mRNAs (A) and mitochondrial ETC components (B) in MscL-G22S-activated SCs were all significantly reduced by inhibition of PI3K. The data shown were representative from four independent experiments. MscL-stretch, MscL-G22S-expressing SCs with mechanical stretching. *p < 0.05, **p < 0.01, ***p < 0.001. [file 12964_2024_1497_MOESM10_ESM.tif]
